# Comparative transcriptome profiling of *Pyropia yezoensis* (Ueda) M.S. Hwang & H.G. Choi in response to temperature stresses

**DOI:** 10.1186/s12864-015-1586-1

**Published:** 2015-06-17

**Authors:** Peipei Sun, Yunxiang Mao, Guiyang Li, Min Cao, Fanna Kong, Li Wang, Guiqi Bi

**Affiliations:** Key Laboratory of Marine Genetics and Breeding (MOE), College of Marine Life Sciences, Ocean University of China, Qingdao, 266003 China; Key Laboratory for Sustainable Utilization of Marine Fisheries Resources, Ministry of Agriculture, Yellow Sea Fisheries Research Institute, Chinese Academy of Fishery Sciences, Qingdao, 266071 China; Institute of Plant Resources, Dalian Nationalities University, Dalian, 116600 China

**Keywords:** *Pyropia yezoensis*, RNA-seq, High temperature stress, Chilling stress, Freezing stress, DGE, Transcriptome

## Abstract

**Background:**

*Pyropia yezoensis* is a model organism often used to investigate the mechanisms underlying stress tolerance in intertidal zones. The digital gene expression (DGE) approach was used to characterize a genome-wide comparative analysis of differentially expressed genes (DEGs) that influence the physiological, developmental or biochemical processes in samples subjected to 4 treatments: high-temperature stress (HT), chilling stress (CS), freezing stress (FS) and normal temperature (NT).

**Results:**

Equal amounts of total RNAs collected from 8 samples (two biological replicates per treatment) were sequenced using the Illumina/Solexa platform. Compared with NT, a total of 2202, 1334 and 592 differentially expressed unigenes were detected in HT, CS and FS respectively. Clustering analysis suggested *P. yezoensis* acclimates to low and high-temperature stress condition using different mechanisms: In heat stress, the unigenes related to replication and repair of DNA and protein processing in endoplasmic reticulum were active; however at low temperature stresses, unigenes related to carbohydrate metabolism and energy metabolism were active. Analysis of gene differential expression showed that four categories of DEGs functioning as temperature sensors were found, including heat shock proteins, H2A, histone deacetylase complex and transcription factors. Heat stress caused chloroplast genes down-regulated and unigenes encoding metacaspases up-regulated, which is an important regulator of PCD. Cold stress caused an increase in the expression of FAD to improve the proportion of polyunsaturated fatty acids. An up-regulated unigene encoding farnesyl pyrophosphate synthase was found in cold stress, indicating that the plant hormone ABA also played an important role in responding to temperature stress in *P. yezoensis*.

**Conclusion:**

The variation of amount of unigenes and different gene expression pattern under different temperature stresses indicated the complicated and diverse regulation mechanism in response to temperature stress in *P. yezoensis*. Several common metabolism pathways were found both in *P. yezoensis* and in higher plants, such as FAD in low-temperature stress and HSP in heat stress. Meanwhile, many chloroplast genes and unigene related to the synthesis of abscisic acid were detected, revealing its unique temperature-regulation mechanism in this intertidal species. This sequencing dataset and analysis may serve as a valuable resource to study the mechanisms involved in abiotic stress tolerance in intertidal seaweeds.

**Electronic supplementary material:**

The online version of this article (doi:10.1186/s12864-015-1586-1) contains supplementary material, which is available to authorized users.

## Background

Because plants lack the ability of locomotion, they are exposed to various environmental stresses. Temperature stress is the most common type of stress to which plants are subjected. Temperature-related stress can occur at (a) temperatures below freezing, (b) low temperatures above freezing, and (c) high temperatures [1]. Stressful temperature conditions can damage the enzymes needed for photosynthesis, respiration, and protein synthesis and so affect the growth and development of plants [2]. Consequently, plants have evolved mechanisms to monitor their environments and to respond with cellular, physiological, and developmental changes to optimize growth and reproductive success.

Intertidal seaweeds inhabit an inherently stressful environment with rapidly changing physical conditions. Seaweed in this zone undergoes extreme environmental changes that include desiccation, osmotic shock, exposure to intense sunlight, and high and freezing temperatures. A better understanding of the mechanisms involved in abiotic stress tolerance in seaweeds could help elucidate their successful survival, reproduction, and distribution in the intertidal region. Transcriptome analysis is an efficient way of achieving this goal. A transcriptome is a complete set of the transcripts in a cell that becomes active at a specific developmental stage or under certain physiological conditions. Transcriptomes provide information that can be used to identify the functional elements of the genome and the molecular constituents of cells and tissues [3]. Digital gene expression (DGE), which is based on the sequencing of genome-wide expression profiles, is an efficient method that can be used to analyze transcriptome data and so identify, quantify, and annotate expressed genes at the genome level even without prior sequence knowledge. This allows for higher confidence in target discovery and pathway studies. Currently, this technique is widely used in higher plant research. In the Chinese bayberry (*Myrica rubra*), it has been used to examine gene expression in developing bayberry fruit. Results showed energy-related metabolism to be enhanced and all genes involved in anthocyanin biosynthesis to be up-regulated during the fruit ripening processes [4]. In *Lycoris sprengeri*, DGE was performed to evaluate differential gene expression between bulbs and bulblets and determine the biological and molecular mechanisms underlying bulb development [5]. In *Vitis vinifera*, it was used to describe how plant transcriptomes change during three developmental stages, post setting, veraison, and ripening [6].

*Pyropia yezoensis* (Ueda), M.S. Hwang & H.G. Choi, one of the most economically important marine crops, is widely cultivated in China, Japan, and Korea, with an annual harvest of more than 1 million tons (fresh weight) and a value of over U.S. $1.5 billion per year (http://www.fao.org/fishery/statistics/en). *P. yezoensis* is naturally distributed in the intertidal zone of the temperate region in the northern hemisphere. In this region, the temperature may change dramatically between seawater and air, especially in the changeover seasons between autumn and winter as well as winter and spring. The thallus of *P. yezoensis* is totally submerged in the water during high tide but exposed to air during low tide. Consequently, the thallus may suffer from the stress of high or low temperature and abrupt temperature changes. This makes this alga an ideal research model for investigations of the mechanisms underlying temperature stress tolerance in intertidal seaweed.

The effect of temperature on algae has been studied. In *Chondrus crispus* it has been reported that plants subjected to high natural stress have more differentially expressed genes and more potential marker genes, and express more antioxidative genes and HSPs. Many of these up-regulated genes are stress genes [7]. *Gracilaria cornea* showed photosynthetic and respiratory responses adapted to oceanic salinities and subtropical to tropical water temperatures [8]. Studies of *Pyropia* have been conducted at the physiological, genomic, and proteomic level. For example, Kayama et al. reported that water temperature can affect the fatty acid composition of *Pyropia* [9]. Choi et al. reported that most of the transcripts produced by *Pyropia tenera* under high-temperature conditions were from the heat shock protein family [10]; Xu et al. conducted a comparative proteomic analysis of *Pyropia haitanensis* in response to high-temperature stress using liquid chromatography-tandem mass spectroscopy and database searching, indicating that the algal blades resisted high-temperature stress by inhibiting photosynthesis and other nonessential metabolic processes [11].

In this paper, gene expression and regulation in response to temperature stresses were examined by performing genome-wide high-throughput transcriptomic sequencing for *P. yezoensis*. In view of the unique intertidal environment, four treatments were used: high-temperature stress, chilling stress, freezing stress and normal temperature. The DGE approach was used to comparatively analyze the expression patterns and characterize the genes that influenced physiological, developmental, and biochemical processes on a genome-wide basis when *P. yezoensis* was subjected to extreme temperature fluctuations. This sequencing dataset and analysis may serve as a valuable resource for identifying the key genes and pathways involved in responses to temperature stresses in *P. yezoensis*. This will lay the foundation for identifying the mechanisms underlying extreme temperature tolerance and may provide information needed for the genetic breeding of high- and low-temperature tolerant varieties.

## Results

### Global transcriptome assembly and annotation

**Figure 1:**
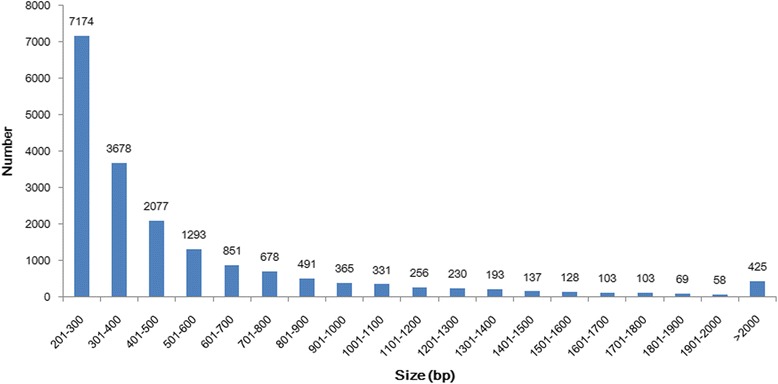
Size distribution of the assembled unigenes.

A global transcriptome was generated using samples from different developmental phases and treatments to obtain as many functional gene transcripts as possible. A total of 1.34 × 10^7^ quality paired-end reads were obtained after filtering out low-quality data (tags containing unknown base N and only adaptor tags). The GC content of the transcriptome was 63.2%. After assembly and annotation, a total of 18,640 unigenes with a mean length of 527 bp and an N50 value of 641 bp were obtained. Unigenes with lengths between 200 and 500 bp were overrepresented, comprising approximately 69.4% of the total number of unigenes; the next most abundant size class was 500–1000 bp, constituting approximately 19.7% of the total unigenes. There were fewer unigenes with lengths of 1–2 kbp and more than 2 kbp, i.e., approximately 8.6% and 2.3% of the total, respectively. The longest unigene was 8372 bp (Figure 1).

BLAST analysis was performed on all 18,640 unigenes using the following databases: NCBI non-redundant protein sequences (Nr), NCBI non-redundant nucleotide sequence (Nt), Swiss-Prot, Kyoto Encyclopedia of Genes and Genomes database (KEGG), Protein family (PFAM), and Clusters of Orthologous Groups of proteins (KOG). There were 10,597 (56.9%) unigenes with homologous sequences in at least one of these databases. Among them, 5937 (31.85%), 4845 (26.0%), and 3225 (17.3%) unigenes were found in NR, Swiss-Prot, and KEGG, respectively (Table 1).

**Table 1:** **Summary of assembly and annotation results for** ***P. yezoensis*** **using Trinity**

**Database**	***Number and ratio of annotated unigenes***
Unigene with annotation	10,597 (56.85%)
Annotated in Nr	5937 (31.85%)
Annotated in KEGG	3225 (17.3%)
Annotated in SwissProt	4845 (25.99%)
Annotated in PFAM	7918 (42.47%)
Annotated in GO	8170 (43.83%)
Annotated in KOG	6372 (34.18%)
Annotated in all databases	638 (3.42%)
Total number of unigenes	18,640 (100%)

### Sequencing and annotation of eight DGE libraries

**Figure 2:**
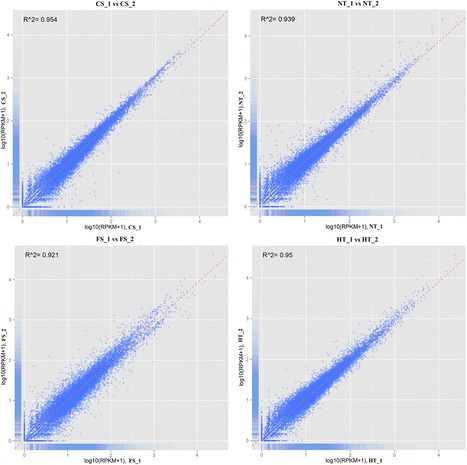
Correlation tests for the replicates. The abscissa represents the value log10 (RPKM + 1) of one duplicate; the ordinate represents the value log10 (RPKM + 1) of the other duplicate. R^2^ is the square of Pearson Correlation Coefficient.

A total of eight RNA samples, generated from two biological replicates of *P. yezoensis* under the four treatments were subjected to RNA-seq (Table 2). Approximately 11.1–12.6 million raw reads were sequenced per sample. After filtering out low-quality data (tags containing unknown base N and only adaptor tags), approximately 10.7–12.1 million clean reads remained in each library. Approximately 73.1–84.5% reads in the eight DGE libraries were mapped to the global transcriptome of *P. yezoensis* (Table 2), suggesting that the transcriptome was a reliable reference. To evaluate the reproducibility of DEG library sequencing, a Pearson correlation analysis was performed for every two replicates. The square of the Pearson correlation coefficient (R^2^) was greater than 0.92, indicating both operational stability and the reliability of the experimental results (Figure 2).

**Table 2:** **Summary of sequencing and mapping results for eight DGE libraries**

**Sample name**	**Raw reads**	**Clean reads**	**Clean bases**	**Error rate (%)**	**Q20 (%)**	**Q30 (%)**	**GC content (%)**	**Total mapped**
NT_1	11,101,544	10,678,262	1.07G	0.06	95.17	86.58	64.04	9,074,411 (84.98%)
NT_2	11,845,613	11,378,840	1.14G	0.06	94.9	85.88	63.6	8,380,392 (73.65%)
HT_1	12,369,188	11,878,032	1.19G	0.06	94.94	86.05	64.38	9,518,267 (80.13%)
HT_2	12,595,064	12,075,828	1.21G	0.06	94.81	85.84	64.58	9,402,614 (77.86%)
CS_1	12,098,964	11,676,535	1.17G	0.06	95.21	86.49	63.97	8,797,628 (75.34%)
CS_2	11,856,911	11,420,971	1.14G	0.06	94.97	85.48	63.54	8,342,733 (73.05%)
FS_8_1	12,320,559	11,705,396	1.17G	0.06	92.15	80.32	63.83	8,788,660 (75.08%)
FS_8_2	12,588,529	12,077,780	1.21G	0.06	94.8	85.79	64.53	9,605,511 (79.53%)

### Global analysis of the unigenes in the four temperature treatment groups

**Figure 3:**
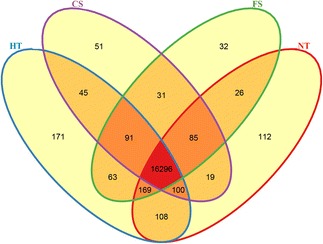
Venn diagram showing the number of expressed unigenes in the NT, HT, CS, and FS treatments. This Venn diagram was drawn using the number of genes expressed (RPKM > 0.3) in the four treatments (FS, HT, CS, and NT).

DESeq analysis was performed to identify the genes that had been differentially expressed between the different temperature treatment groups. A total of 17,399 expressed unigenes were obtained, of which 16,296 were found in all samples (Figure 3). The comparison showed 171 unigenes to be unique to the high-temperature treatment group, 51 to the chilling group, and 32 to the freezing group.

**Figure 4:**
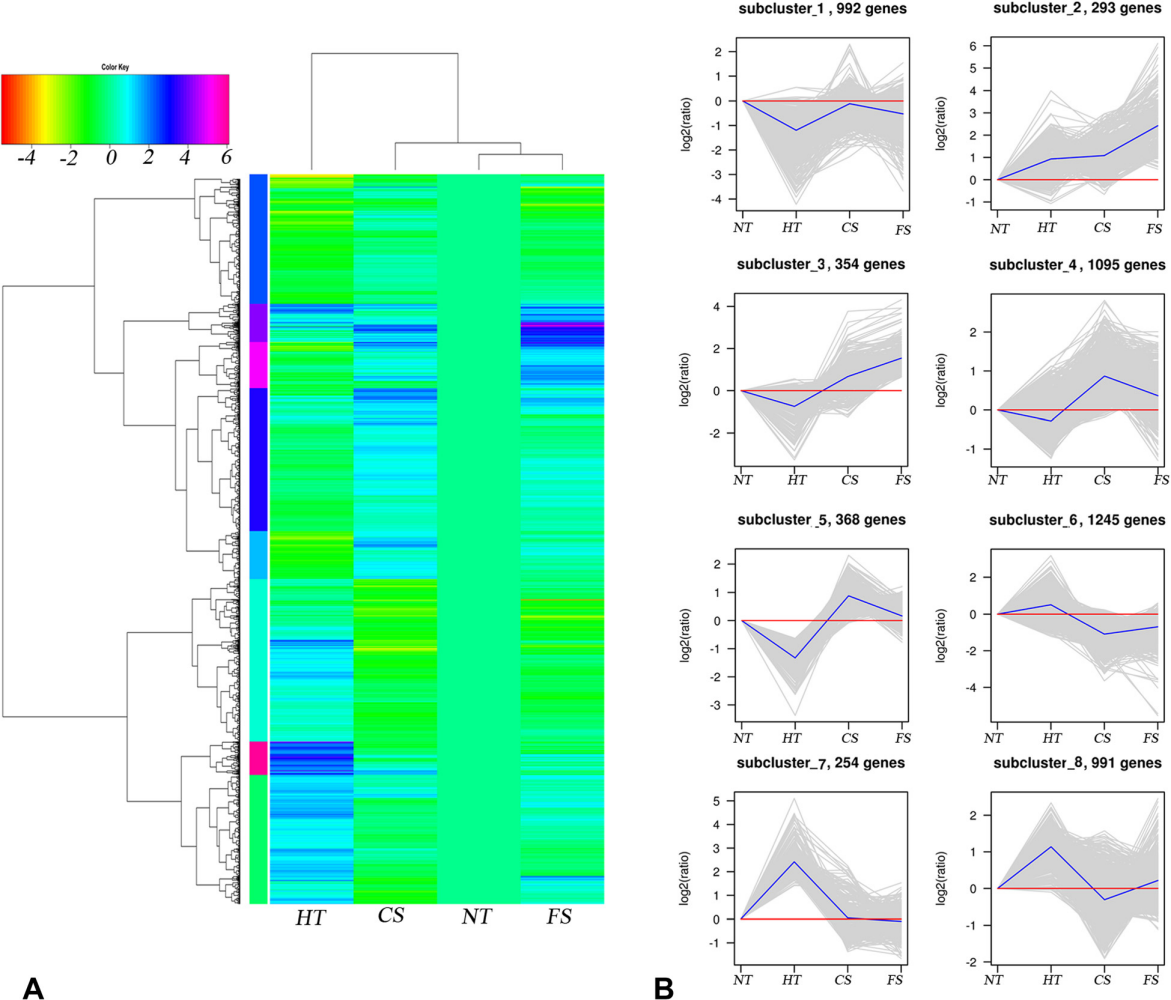
Clustering analyses of DEGs in the four temperature treatments. Clusters were obtained using the hierarchical-means method based on the 5592 DEGs (the intersection set of the DEGs in the four treatments). **(A)** Heatmap of hierarchical clustering. Each column represents a treatment, and each row represents a unigene. Differences in expression were shown in different colors. Negative numbers indicate down-regulated and positive number means up-regulated. **(B)** Hierarchical clustering figure of differentially expressed genes. The “x” axis represents the treatments. The “y” axis represents the value of the relative expression level (log2 (HT, CS, FS/ NT)). In the subcluster, the gray line represents the value of the relative expression levels, the blue line represents the mean value of the relative expression level, and the red line is the reference. The subclusters in Figure **B** represent the models in Figure **A** from the top down.

A hierarchical cluster was used to determine the profiles of the differentially expressed unigenes among the four temperature stress treatments (Figure 4). Hierarchical clustering showed that the differentially expressed unigenes could be divided into 8 subclusters based on the modulation of their expression, representing 8 different expression models (Figure 4). Unigenes with the same or similar expression patterns were gathered in the same cluster. The overall pattern of transcript changes in the freezing temperature treatment at −8°C and the chilling temperature at 0°C were similar to that of the normal temperature at 8°C. The pattern under the high temperature treatment at 24°C was markedly different from that of the other three treatment groups (Figure 4A). The results clearly indicate that *P. yezoensis* used different mechanisms in response to high- and low-temperature stresses, but it used similar mechanisms in response to the chilling and freezing treatments.

In subcluster 1, the unigenes were found to be closely related to translation of genetic information. These included genes related to the pathway of ribosome and RNA transport. In subcluster 2, the pathway associated with carbohydrate metabolism and energy metabolism were significantly enriched, including genes for the pentose phosphate pathway, glycolysis and carbon fixation in photosynthetic organisms. In subcluster 3, the unigenes related to the synthesis and decomposition of carbohydrates were affected in a manner similar to those in subcluster 2, except that there were more unigenes related to amino acid metabolism. In subcluster 4, subcluster 5, and subcluster 8, the unigenes were not concentrated but rather related to many processes (see Additional file 1). In subcluster 6, the unigenes were up-regulated in response to high-temperature stress, and the pathway associated with replication and repair of DNA were significantly enriched, showing the same expression trends as in subcluster 7. These unigenes were closely related to protein processing in the endoplasmic reticulum.

### Number of differential expression unigenes

**Figure 5:**
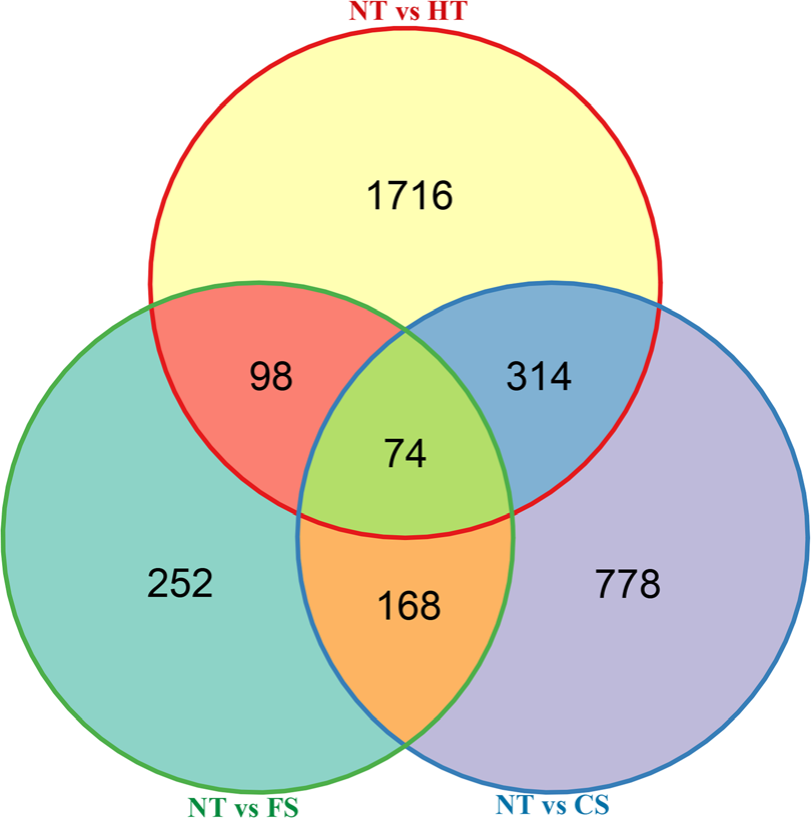
Venn diagram of DEGs under the control and temperature-stressed conditions. This Venn diagram was drawn using the number of different expression genes in the three stress treatments (FS, HT, and CS) relative to the control group (NT).

Differentially expressed unigenes with a q-value ≤ 0.05 [12] between pairwise combinations of the treatments were identified. A total of 2202, 1334, and 592 significantly altered unigenes were detected between NT and HT, NT and CS, and NT and FS, respectively. A total of 74 unigenes showed expression different from NT in all three treatment groups (Figure 5).

### Gene Ontology functional analysis of DEGs

**Figure 6:**
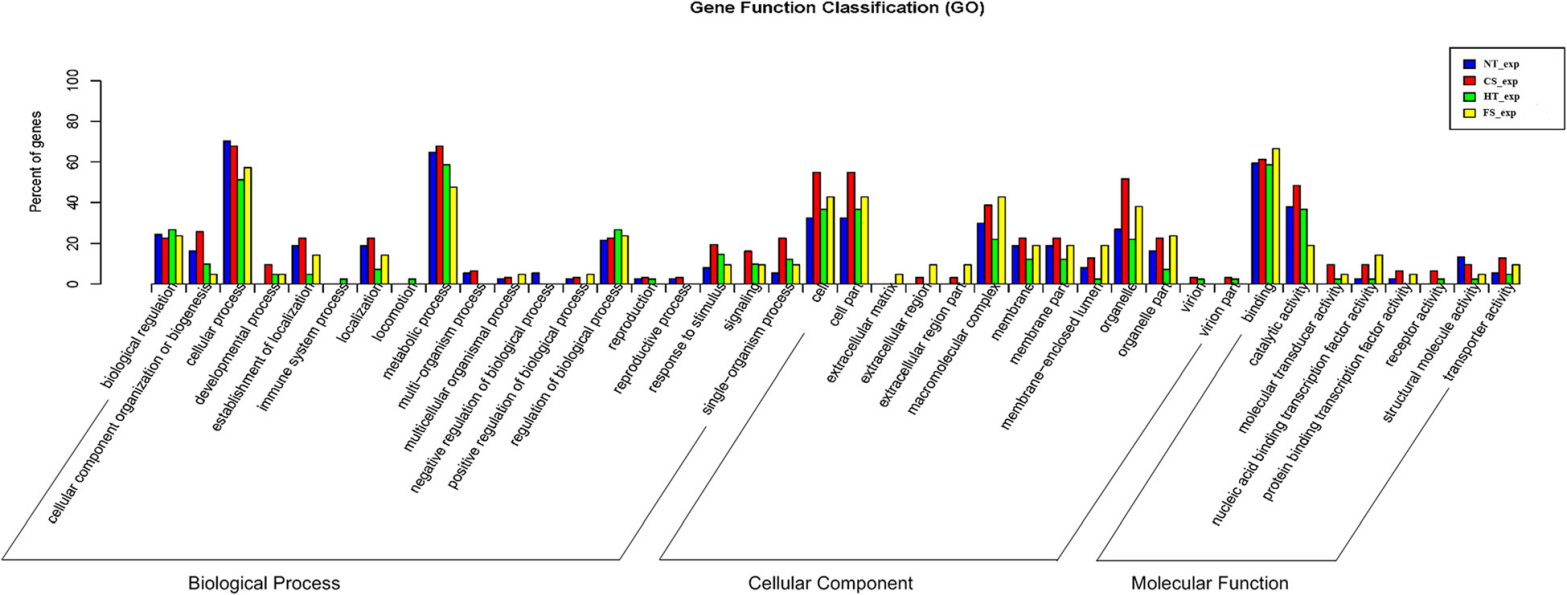
Comparative distribution of the GO terms in the four treatment groups.

Gene Ontology functional analysis of DEGs was performed to describe the properties of genes and their products in *P. yezoensis* subjected to different temperature stresses. For the biological process category, the two mostly highly represented terms among the 19 level-2 categories were cellular process and metabolic process; for the cellular component category, the mostly highly represented terms among the 13 level-2 categories were cell, cell part, macromolecular complex and organelle; for the molecular function category, the two mostly highly represented terms among the 8 level-2 categories were binding and catalytic activity (Figure 6).

### DEGs in response to chilling treatment

**Figure 7:**
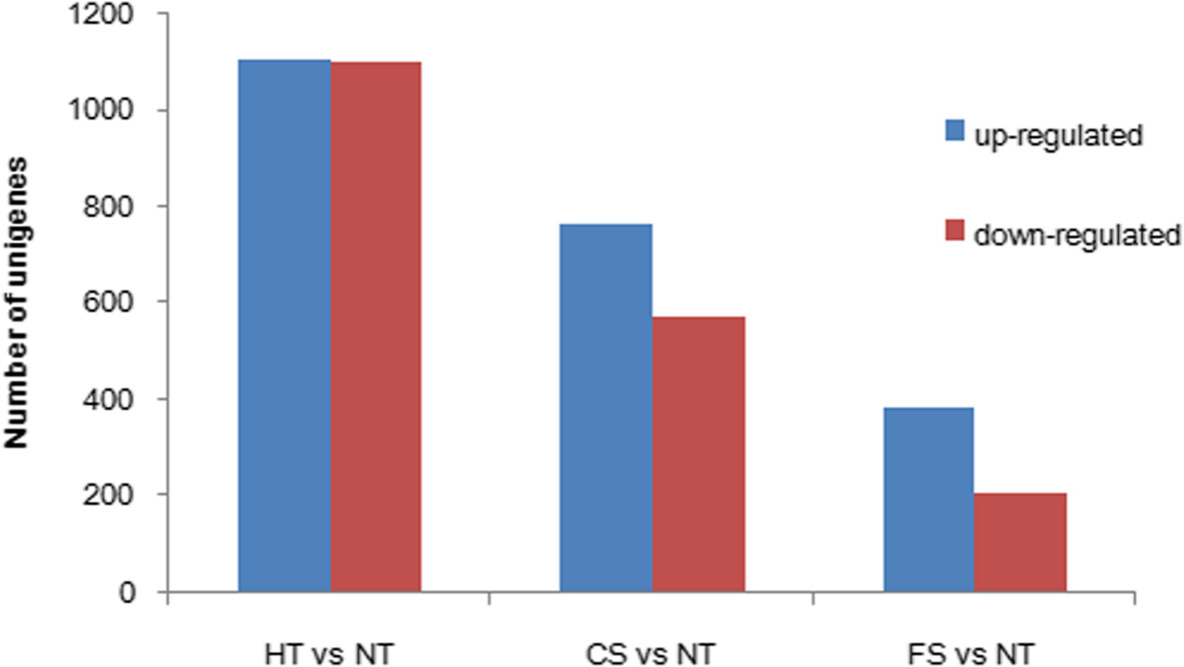
Number of DEGs in the different temperature stress groups.

A total of 1334 differentially expressed unigenes were identified in CS, relative to NT. These included 762 up-regulated unigenes and 572 down-regulated unigenes (Figure 7). The top 100 down- and up-regulated genes are shown in Additional files 2 and 3. Based on KEGG analysis, the up-regulated unigenes were highly enriched in ribosome biogenesis in eukaryotes, alanine, aspartate and glutamate metabolism, pyrimidine metabolism, and biosynthesis of unsaturated fatty acids. The down-regulated unigenes were highly enriched in aminoacyl-tRNA biosynthesis and porphyrin and chlorophyll metabolism. GO enrichment showed there to be 1 and 7 significantly enriched GO terms in the up- and down-regulated unigenes, respectively (see Additional file 4).

In higher plants, increased synthesis of polyunsaturated fatty acids (predominantly trienoic) occurs in response to chilling stress. DEG analysis revealed 12 fatty acid desaturase (FAD) unigenes among the 762 up-regulated unigenes, but no FAD unigenes were found among the down-regulated unigenes. Among the 1334 DEGs, unigenes encoding glutathione S-transferase, oxidoreductase, and thioredoxin were found. These promote chilling tolerance by maintaining cell redox homeostasis. Two categories of DEGs functioning as temperature sensors were analyzed, both histone deacetylase complex and transcription factor. Results showed an up-regulated unigene encoding the histone acetyltransferase complex and a down-regulated unigene encoding the histone deacetylase complex. 13 unigenes encoding transcription factor that included basic leucine zipper (bZIP), upstream activation factor (UAF), and myeloblastosis oncogene were differentially regulated.

### DEGs in response to freezing treatment

A total of 592 differentially expressed unigenes were found in FS, relative to NT; 383 unigenes were up-regulated and 209 unigenes were down-regulated (Figure 7). The top 100 down- and up-regulated genes are shown in Additional files 5 and 6. There were fewer DEGs in FS than in CS and HT. It can be inferred that, under freezing conditions, many physiological functions are suspended and the expression levels of the unigenes were maintained at a normal level. Among the up-regulated unigenes, there were 7 *FAD* unigenes and one unigene encoding farnesyl pyrophosphate synthase. There were also many unigenes encoding glutathione S-transferase, oxidoreductase, thioredoxin, and the histone acetyltransferase complex, which promote chilling tolerance, like the unigenes found in CS. As noted in the global analysis of the gene expression component, FS has a pattern of expression similar to that of CS. GO enrichment analysis showed there to be 3 significantly enriched GO terms among the up-regulated unigenes and 26 significantly enriched GO terms among the down-regulated unigenes (see Additional file 7).

### DEGs in response to heat treatment

A total of 2202 differentially expressed unigenes were identified in HT, relative to NT; 1103 unigenes were up-regulated and 1099 unigenes were down-regulated (Figure 7). The top 100 down- and up-regulated genes are shown in Additional files 8 and 9. KEGG analysis showed that the up-regulated DEGs were highly enriched in protein processing in endoplasmic reticulum and the down-regulated unigenes were enriched in ribosome biogenesis in eukaryotes, one carbon pool by folate, alanine, aspartate, and glutamate metabolism, and RNA transport. GO enrichment analysis showed that 116 GO terms were significantly enriched in the down-regulated unigenes, but among the up-regulated unigenes, no GO terms were enriched (see Additional file 10).

In this study, three categories of DEGs functioning as temperature sensors were found, including heat shock proteins (HSPs), H2A, and transcription factors. Among the up-regulated unigene pools, there were 18 well-characterized *HSP*s (i.e., 3 Hsp20, 2 Hsp90, 4 Hsp60 and 9 Hsp70). In addition, 4 up-regulated unigenes encoding H2A, which is believed to be a temperature sensor, were found. An additional 22 transcription factor (*TF*) unigenes were differentially regulated in HT and NT, including bZIP, C-repeat binding factor (CBF), *TFIIB*, and *UAF*. A multiprotein bridging factor 1 (*MBF1*) was up-regulated. Among the top 20 down-regulated unigenes, 15 were chloroplast genes. Four unigenes encoding metacaspase were found to be up-regulated under high temperature stress which was consider to play an important role in programmed cell death.

### Validation of RNA-Seq-based gene expression

**Figure 8:**
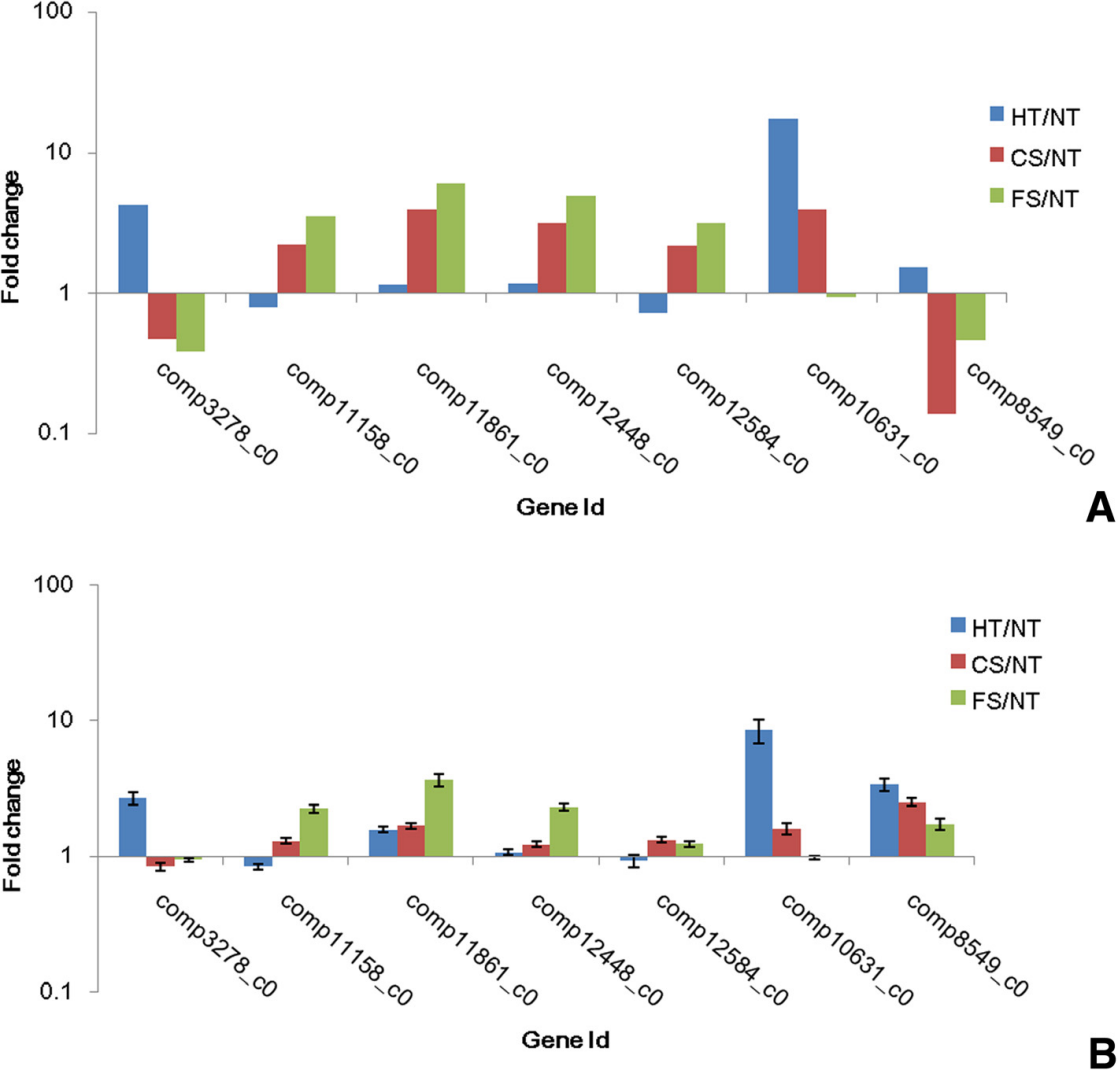
RT-PCR analysis of 7 randomly selected unigenes. ((A) Gene expression data for DGE analysis. The fold changes of the genes were calculated as the log2 vaule of HT/NT, CS/NT and FS/NT and shown on the y-axis. (B) The qRT-PCR analysis of gene expression data. Expression ratios of these genes in HT, CS and FS were compared to NT, respectively).

To validate the expression profiles obtained by RNA-Seq, RT-PCR was performed on seven genes selected at random with high or low expression levels. Expression comparisons were performed between HT and NT, CS and NT, and FS and NT by RT-PCR. For all of the genes, the trend in RT-PCR expression was in agreement with the RNA-Seq data except for comp8549_c0 (Figure 8).

## Discussion

### Role of unsaturated fatty acids under low temperature stress conditions

**Figure 9:**
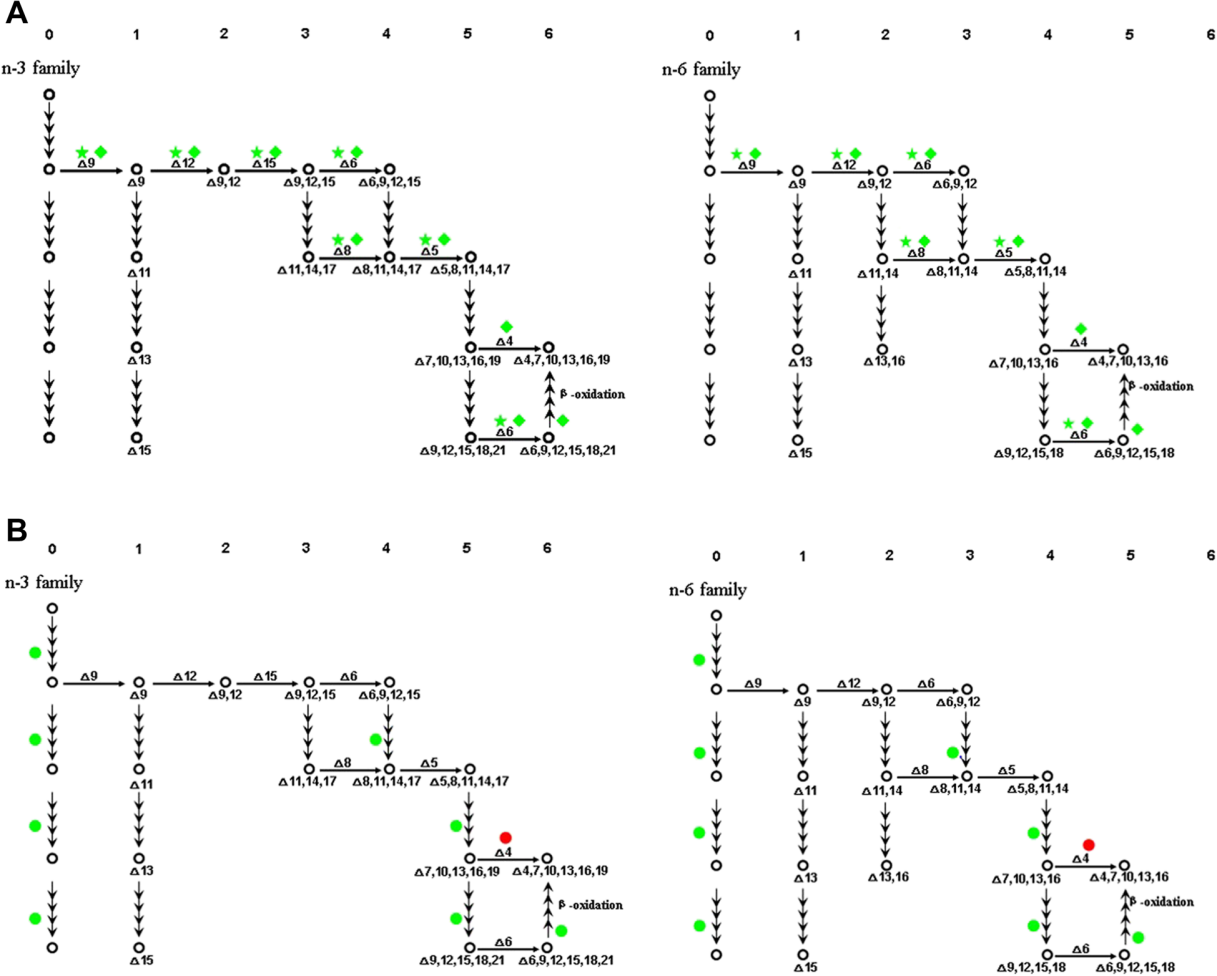
Differential gene expression in the biosynthesis pathway of unsaturated fatty acids in P. *yezoensis*. (Green represents up-regulated unigenes; red represents down-regulated unigenes. ★ represents unigenes differentially expressed between NT and CS; ◆ represents unigenes differentially expressed between NT and FS. ● represents unigenes differentially expressed between NT and HT).

In general, plants regulate the proportion of polyunsaturated fatty acids to change membrane fluidity in response to temperature stress. In both yeast and cyanobacteria, it has been hypothesized that temperature-mediated alterations of membrane fluidity may themselves be the primary temperature sensing event. It has been speculated that the same might be true in higher plants [13]. In this study, a total of 30 unigenes encoding fatty acid desaturases were found, in which 12 unigenes belonging to 7 diverse types were differentially expressed (Table 3). In higher plants, it has been reported that cold tolerance is closely correlated to the level of unsaturated fatty acids in phosphatidylglycerol from the chloroplast membrane, especially in the sn-1 position [14]. Plants synthesize 18:2 n-6 and 18:3 n-3 from saturated fatty acid 18:0; these are catalyzed by soluble delta-9 acyl-ACP, membrane-bound delta-12 fatty acid and ω3 desaturases, respectively [15]. Delta-9 fatty acid desaturase catalyzes the formation of the initial double bond between the 9th and 10th carbons of palmitoyl (16:0) and stearoyl (18:0) coenzyme A (CoA) substrates to produce 16:1 and 18:1 fatty acids, respectively [16]. Delta-15 fatty acid desaturase is located in plastids and catalyzes the introduction of a double bond into 18:2 n-6 esterified in the sn-1 position of glycerolipids [17]. In the CS and FS treatments, all 10 *FAD* were up-regulated, promoting the synthesis of fatty acid desaturases, thereby increasing membrane fluidity, which results in acclimation to chilling stress in the intertidal zone. In the high-temperature treatment group, 2 *FAD* were down-regulated. In the KEGG database, 12 unigenes were successfully annotated to the pathway of unsaturated fatty acid biosynthesis (Figure 9). In the high-temperature treatment group, in addition to the 2 down-regulated *FADs*, the unigene comp704_c0 encodding 3-hydroxy acyl-CoA dehydratase was up-regulated which takes part in fatty acid elongation.

**Table 3:** **Differentially expressed unigenes encoding fatty acid desaturase in CS and FS relative to NT**

**Gene ID**	**Annotation**	**CS**	**FS**
comp11158_c0	delta-5 fatty acid desaturase	↑	↑
comp11646_c0	delta-5 fatty acid desaturase	↑	↑
comp12448_c0	delta-12 fatty acid desaturase	↑	↑
comp12584_c0	delta-15 fatty acid desaturase	↑	↑
comp11861_c0	delta-6 fatty acid desaturase	↑	↑
comp11916_c0	delta-9 fatty acid desaturase	↑	↑
comp13086_c0	fatty acid desaturase	- -	- -
comp6703_c0	fatty acid desaturase	↑	↑
comp8809_c0	fatty acid desaturase	↑	- -
comp8918_c0	delta-4 fatty acid desaturase	- -	- -
comp8636_c0	delta-8 fatty acid desaturase	↑	↑
comp11712_c0	delta-4 fatty acid desaturase	- -	↑

### HSP in response to temperature stresses

It is well established that HSP contributes greatly to cell protection through the folding and translocation of nascent proteins, the refolding of denatured proteins, and the disassembly of already formed protein aggregates under both stress and non-stress conditions [18-21]. Based on subunit sizes, HSP can be divided into 5 groups, HSP100, HSP90, HSP70, HSP60, and small HSP. In this study, a total of 56 unigenes were found to encode HSP. The expression level of the most of the unigenes was not significantly different, while their expression levels were high (see Additional file 11). In addition, *HSP70*, *HSP90*, *HSP60*, and *HSP20* were found to significantly differently expressed as a result of the temperature treatment (Table 4). Recently, HSP70 has attracted widespread attention; this protein plays a vital role in the transport of nascent proteins across membranes into organelles, the folding of newly translated proteins, the repair of misfolded proteins, and targeting damaged proteins for degradation [20]. When plants suffer from stress, HSP70s are up-regulated, participate in the refolding of denatured proteins, maintain cell homeostasis, and protect organisms from damage [22]. Nine unigenes encoded HSP70; six were up-regulated and three were down-regulated (Table 4). HSP60 has been shown to function as a chaperonin in the assembly of mitochondrial enzyme complexes composed of proteins encoded by nuclear genes and imported from the cytosol [23]. All 4 *HSP60* genes were down-regulated in both in HT and FS. In general, the *HSP* unigenes did not exhibit regular changes. It is here assumed that the HSP unigenes maintain high expression levels prior to frequent habitat changes to allow the organism to acclimate to the intertidal environment.

**Table 4:** **Variation of HSP unigene expression under different treatments compared to NT**

**Gene ID**	**Annotation**	**HT**	**CS**	**FS**
comp10260_c0	HSP70 superfamily	- -	↑	- -
comp11228_c0	HSP70 superfamily	- -	- -	↓
comp11281_c0	HSP70 superfamily	↓	- -	- -
comp12891_c0	HSP70 superfamily	↑	- -	- -
comp13402_c0	HSP70 superfamily	- -	↑	- -
comp2728_c0	HSP70 superfamily	↑	- -	- -
comp2735_c0	HSP70 superfamily	↑	- -	- -
comp2752_c0	HSP70 superfamily	- -	↓	- -
comp6578_c0	HSP70 superfamily	- -	↑	- -
comp2723_c0	HSP20 family protein	↑	- -	- -
comp10631_c0	HSP20 family protein	↑	↑	- -
comp38018_c0	HSP20 family protein	↓	- -	↓
comp12212_c0	Mitochondrial chaperonin, Cpn60/Hsp60p	↓	- -	- -
comp30324_c0	Mitochondrial chaperonin, Cpn60/Hsp60p	↓	- -	↓
comp6347_c0	Mitochondrial chaperonin, Cpn60/Hsp60p	- -	- -	↓
comp7933_c0	Mitochondrial chaperonin, Cpn60/Hsp60p	↓	- -	↓
comp3278_c0	Heat shock protein 90	↑	- -	- -
comp9949_c0	Heat shock protein 90	- -	- -	↓

### Histones in response to temperature stresses

Histones are basic proteins that package DNA into nucleosomes, and histone gene expression is closely correlated with the cell cycle and cell proliferation [24]. It has been reported that histone genes are repressed by cold stress [25]. Some researchers believe that, at cooler temperatures, H2A.Z occupancy represses gene expression by creating a physical block to transcription or by preventing the binding of complexes that activate transcription [26]. CS treatment showed less histone expression than NT, but FS treatment showed no differently expressed unigenes that encoded histone. A unigene encoding histone acetyltransferase complex was found to be up-regulated and a unigene encoding histone deacetylase complex that was found to be down-regulated (Table 5). Histone acetylation and deacetylation processes occur at specific lysine residues within the N-terminal. One theory suggests that ethanoyl is electronegative and can neutralize the electropositivity of histones. Consequently, ethanoyl can reduce the affinity of histones and DNA, which is electronegative. In this way, the acetylation of histone can lose nucleosomes in order to activate transcription. In the heat treatment, 4 histone unigenes including *H2A, H2B, H3*, and *H4* were found to be up-regulated.

**Table 5:** **Variation in the expression of unigenes related to histone and acetylation in HT, CS, and FS relative to NT**

**Gene ID**	**Annotation**	**HT**	**CS**	**FS**
comp50541_c0	Histone acetyltransferase complex	- -	↑	↑
comp46060_c0	Histone deacetylase complex	- -	↓	- -
comp11234_c0	Histone H2A	↑	- -	- -
comp11479_c0	Histone H4	↑	- -	- -
comp12414_c0	Sperm histone P2	- -	↓	- -
comp12441_c0	Histone H3	↑	- -	- -
comp9210_c0	Histone H2B	↑	- -	- -

### Multi-protein bridging factor and transcription factors

Multiprotein bridging factor 1 (MBF1) is a highly conserved transcriptional coactivator involved in the regulation of diverse processes, such as endothelial cell differentiation, histidine metabolism, hormone-regulated lipid metabolism, and central nervous system development [27-29]. MBF1 functions up-stream of salicylic acid, trehalose, and ethylene during high-temperature stress by causing the accumulation of numerous transcription factors and transcription products of signal transduction genes [30]. In the current study, the expression of *MBF* was higher in HT but remained unchanged under cold stress (both FS and CS), which suggests that cold stress had little effect on the expression patterns of PyMBF1. A similar result was reported in a previous study which found that PyMBF1 transcripts are up-regulated in *P. yezoensis* cells during exposure to oxidative and heat stresses, and that the heat activation of PyMBF1 requires membrane fluidization [31]. In addition, 5 Zn-finger protein unigenes were up-regulated. These are believed to be controlled by MBF1 at the transcriptional level during high-temperature stress. In *Arabidopsis thaliana*, MBF1c acts as a transcriptional regulator. It binds DNA and controls the expression of 36 different transcripts during heat stress, including the important transcriptional regulator DRE-binding protein 2A (DREB2A), two heat shock transcription factors (HSFs), and several zinc finger proteins [32]. The current work suggests that *PyMBF1* plays a role in the oxidative and heat stress response pathways in *P. yezoensis*.

Transcription factors, as key regulators of gene expression can be found in any organism. These factors act downstream of signaling cascades related to biological and environmental stimuli. In this study, transcription factors played an important role in response to temperature stresses. Five up-regulated *bZIP* unigenes were detected in HT and two down-regulated *bZIP* unigenes were detected in CS. The expression of *MYB* was higher in CS. Two *TF* families, *bZIP* and *MYB*, are involved in ABA signaling and its gene activation, which plays a vital role in plant stress responses [33]. MYB proteins are key to regulatory networks controlling development, metabolism, and responses to biotic and abiotic stresses [34]. It has been suggested that MYB15 is part of a complex network of transcription factors that control the expression of *CBFs* and other genes in response to cold stress [35].

### Programmed cell death in response to high-temperature stress

In unicellular organisms, programmed cell death (PCD) is a protective action that keeps their cellular best adapted to survival under stress conditions. It may also serve as a possible mechanism for managing cell cycles and differentiation [36]. Caspases are cysteine proteases that are important regulators of PCD in animals. Plant genomes do not contain structural homologs of caspases. Instead, they encode several related proteins, called metacaspases. These are also present in other organisms such as fungi, parasitic protozoa, and some bacteria [37]. Metacaspases are suggested to be the ancestors of metazoan caspases, and plant metacaspases have previously been shown to be genuine cysteine proteases that automatically process their substrates in a manner similar to that of caspases [38]. Many studies have been performed to explore the role of metacaspases in PCD. In *Arabidopsis*, metacaspases AtMC1 and AtMC2 were found to mediate PCD [39]. In yeast cells, metacaspases are involved in PCD when the yeast cells suffer different extracellular stresses such as viral infections and heat shock [40]. Up until now, there have been only a few studies on the relationship between metacaspases and PCD in algae in response to temperature, For example, shifting *Symbiodinium microadriaticum* from 27 to 32°C resulted in an increase in mortality, an increase in caspase 3-like activity, and an increase in nitric oxide (NO) production [41]. In the current work, four unigenes encoding metacaspase were found to be up-regulated under high-temperature stress, but none were found in other treatment groups, from which it can be inferred that the overexpression of the genes encoding metacaspases played an important role in *P. yezoensis* acclimation to heat stress. It has been reported that PCD is a central theme during plant reproductive development, and precise control of PCD execution, or its prevention, are intimately linked with successful plant reproduction [42]. Sexual reproduction could be accelerated in an elevated temperature and light climates; in *Sargassum horneri* it occurred at least 3 months earlier than in the wild [43]. This inspired the present investigation of the expression of genes encoding metacaspases activated under high-temperature stress conditions to regulate the progress of development, accelerating the formation of generative cell in *P. yezoensis*.

### Chloroplast genes in response to high-temperature stress

Under high-temperature treatment conditions, the chloroplast genes encoding 3-oxoacyl-acyl-carrier-protein synthase 3, photosystem I subunit XI, photosystem II 47 kDa protein, ribosomal protein L19, and ribulose-1,5-bisphosphate carboxylase were significantly down-regulated relative to the 8°C group, indicating that less photosynthesis was taking place. Photosystem II (PSII) is one of the most thermosensitive components of photosynthesis and PSI activity is much more heat stable than PSII. The two factors that make PSII electron transport most susceptible to heat stress are (i) increases in the fluidity of thylakoid membranes at high temperature which causes PSII light harvesting complexes to become dislodged from the thylakoid membrane and (ii) dependence of PSII integrity on electron dynamics [44]. Research on *P. tenera* may provide support for the current finding that the gross photosynthesis for the gametophytes of *P. tenera* were determined over a temperature range of 8–34°C. This showed that the gross photosynthetic rates of 46.3 μmol O_2_ mg_chl-a_^−1^ min^−1^ was highest at 9.3 (95% Bayesian credible interval (BCI): 2.3–14.5°C) [45]. It was here assumed that *P. yezoensis* resists high-temperature stress by inhibiting photosynthesis, as has been found in *P. haitanensis* [11].

### Role of hormones in response to temperature stress in *P. yezoensis*

A growing body of evidence suggests that plants use endogenous hormones to couple their growth rate to temperature rather than subject themselves to temperature-induced inhibition of growth [46]. Although abscisic acid (ABA) contains 15 carbon atoms, it is formed in two different ways, directly from the C_15_ sesquiterpene precursor farnesyl diphosphate (FDP), which is the direct pathway, and formed by cleavage of C_40_ carotenoids originating from the MEP pathway, which is considered the indirect pathway [47]. In *Pyropia*, abscisic acid has been detected using liquid chromatography–tandem mass spectrometry [48]. Here, transcriptome data were searched for unigenes related to the biosynthesis of abscisic acid to determine which pathway *P. yezoensis* took to synthesize abscisic acid. There was one unigene encoding farnesyl pyrophosphate synthase, which acts in the direct pathway, but no unigenes related to the indirect pathway were found, not even 9-cis-epoxycarotenoid dioxygenase (*NCED*), which cleaves carotenoid precursors to produce xanthoxin, which can subsequently be converted into ABA [49]. For this reason, it was inferred that the direct pathway was active in *P. yezoensis*, as in the green algae *Dunaliella* [50] and in fungi, *Cercospora cruenta* [51]. This is not the preferred pathway of category higher plants, however [52]. In addition, the unigene encoding farnesyl pyrophosphate synthase was up-regulated in CS. From this, it was inferred that the concentration of abscisic acid increased to acclimate to temperature in FS. In many studies, abscisic acid biosynthesis was found to be an essential requirement for cold acclimatization and acquired thermotolerance [53]. The overexpression of *ABSCISIC ACID INSENSITIVE 3* (*ABI3*) confers increased freezing tolerance in *Arabidopsis* [54].

## Conclusions

This study has demonstrated the usefulness of the digital gene expression approach to identify the differentially expressed genes of *P. yezoensis* under different temperature treatments. First, in response to high temperature stress, *P. yezoensis* showed a constitutive gene expression profile different from that of other treatments, but the two low temperature stresses (i.e., chilling and freezing stress) were similar. Under heat stress, unigenes related to replication and repair of DNA and protein processing in endoplasmic reticulum showed an up-regulated trend. More unigenes related to carbohydrate metabolism and energy metabolism were up-regulated under low-temperature stress conditions than at normal temperatures. The mechanisms involved in temperature response were demonstrated from the following perspectives: membrane fluidity, histones, PCD, photosynthesis, and transcription factors. A large number of genes of unknown function showed significant variations in expression under temperature stress, providing a new and valuable clue for future research into mechanisms by which *P. yezoensis* tolerates the unique environment of the intertidal zone.

## Methods

### Plant materials

To eliminate the interference caused by genotypic differences, a lab-cultured pure line PY4-7 of *P. yezoensis* was used in these experiments. The gametophytes of this pure line were generated from the clonal cultivation of a single somatic cell that originated from a farmed thallus, which was cultured in bubbling natural seawater with Provasoli’s enrichment solution medium (PES) under 50 μmol photons m^−2^ s^−1^ at 8 ± 1°C and a 12:12 light:dark (L:D) photoperiod. Sporophytes were generated from the self-fertility of the pure line and were cultured in bubbling natural seawater with PES under 20 μmol photons m^−2^ s^−1^ at 20 ± 1°C and a 12:12 light:dark (L:D) photoperiod.

### Experimental design and sampling

The material included the samples from different developmental phases and treatments (Table 6). After harvesting and weighing, the samples were immediately frozen in liquid nitrogen [55].

**Table 6:** **Samples used to construct the global transcriptome**

Phase	Treatment conditions
Sporophytes	
Free-living conchocelis filamentous	Natural seawater with Provasoli’s enrichment solution medium (PES),18°C, 24 μmol photos m^−2^ s^−1^, light:dark=12:12
Free-living conchosporangium	Natural seawater with Provasoli’s enrichment solution medium (PES), 24°C, 24 μmol photos m^−2^ s^−1^, light:dark=12:12
Gametophytes	
Thallus	Natural seawater, 9°C, 24 μmol photos m^−2^ s^−1^, 4 h
Thallus	Natural seawater added with deionized water of equal volume, 9°C, 24 μmol photos m^−2^ s^−1^, 4 h
Thallus	Natural seawater added with 33 g NaCl per liter, 9°C, 24 μmol photos m^−2^ s^−1^, 4 h
Thallus	Natural seawater, 9°C, 1500 μmol photos m^−2^ s^−1^, 4 h
Thallus	Natural seawater, 9°C, dark, 4 h
Thallus	--(drought), 4°C, dark, 4 h

For DGE analysis, four temperature treatments were set up: normal temperature (NT, 8°C), high temperature (HT, 24°C), chilling stress (CS, 0°C), and freezing stress (FS, −8°C). Two biological replicates were used in each treatment. After 6 h of treatment, all samples were collected and stored in liquid nitrogen.

### RNA isolation

Total RNA was extracted from the thallus using the Plant RNA Kit (Omega, U.S.) in accordance with the manufacturer’s instructions. RNA degradation and contamination were monitored on 1% agarose gels. The purity was evaluated using a NanoPhotometer spectrophotometer (IMPLEN, CA, U.S.). RNA concentration was measured using a Qubit RNA Assay Kit and Qubit 2.0 Fluorometer (Life Technologies, CA, U.S.). RNA integrity was assessed using an RNA Nano 6000 Assay Kit and the Bioanalyzer 2100 system (Agilent Technologies, CA, U.S.).

### Library preparation for transcriptome and DGE sequencing

A total of 6 μg RNA per sample was used as input material for the RNA sample preparation. All nine RNA samples (including a pooled RNA sample for transcriptome sequencing and eight RNA samples for DGE sequencing) had RIN values above 7.0. Sequencing libraries were generated using an Illumina TruSeq RNA Sample Preparation Kit (Illumina, San Diego, CA, U.S.) in accordance with the manufacturer’s recommendations. Eight index codes were added to attribute sequences to each sample. Briefly, mRNA was purified from total RNA using poly-T oligo-attached magnetic beads. Fragmentation was carried out using divalent cations at elevated temperatures in Illumina proprietary fragmentation buffer. First-strand cDNA was synthesized using random oligonucleotides and SuperScript II (Invitrogen). Second-strand cDNA synthesis was subsequently performed using DNA Polymerase I and RNase H. Remaining overhangs were converted into blunt ends via exonuclease/polymerase activities and then enzymes were removed. After adenylation of 3′ ends of DNA fragments, Illumina PE adapter oligonucleotides were ligated to prepare for hybridization. To select cDNA fragments of 200 bp in length, the library fragments were purified using an AMPure XP system (Beckman Coulter, Beverly, U.S.). DNA fragments with ligated adaptor molecules on both ends were selectively enriched using Illumina PCR Primer Cocktail in a 10-cycle PCR reaction. Products were purified (AMPure XP system) and quantified using an Agilent high-sensitivity DNA assay and Agilent Bioanalyzer 2100 system. Clustering of the index-coded samples was performed on a cBot Cluster Generation System using a TruSeq PE Cluster Kit v3-cBot-HS (Illumia) according to the manufacturer’s instructions. After cluster generation, the libraries were sequenced on an Illumina Hiseq 2000 platform. The sample for transcriptome was sequenced with 100 bp pair end reads. The other eight libraries for DGE were sequenced with 100 bp single end reads for DGE analysis.

#### Quality control

Raw data (raw reads) of fastq format were first processed through in-house Perl scripts. In this step, clean data (clean reads) were obtained by removing reads containing adapter, reads in which more than 10% of the bases were unknown,and low quality reads (where more than 50% of bases in a read had a quality value Q ≤5) from the raw data. The Q20, Q30, and GC content and sequence duplication level of the clean data were calculated. All the downstream analyses were based on clean, high-quality data.

### De novo assembly and annotation

De novo transcriptome assembly was performed using the short-reads assembly program, Trinity (v2012-10-05) with min_kmer_cov set to 2 and all other parameters set default [56]. Using pair-end reads, we detected contigs from the same transcript as well as the distances between these contigs. Next, we used Trinity to connect the contigs and obtain sequences that could not be extended on either end, known as unigenes. The optimal assembly results were chosen according to the assembly evaluation. Then the clustering analysis was performed to achieve a unigene database which comprised the potential alternative splicing transcripts.

After clustering, the unigenes were divided into two classes: clusters and singletons. Finally, BLASTx alignment (E-value < 0.00001) was performed between unigenes and the protein databases, including Nr, Swiss-Prot, KEGG, and KOG. The best alignment results were used to decide the sequence direction of unigenes.The unigenes were compared against those in the NCBI Nr and Nt database and Swiss-Prot database using BLAST 2.2.27+ with an E-value of 1e-10, 1e-5, and 1e-5, respectively. Gene names were assigned to each unigene based on the best BLAST hit (highest score). The unigene sequences were also aligned to the KOG database to predict and classify functions using BLAST 2.2.27+ with an E-value of 1e-3. The unigenes sequences were searched against PFAM database to predict functional domain and protein family using Hmmerscan (HMMER 3.0 package) with an inclusion E value of 0.01.

To annotate the unigenes with GO terms describing biological processes, molecular functions and cellular components, the Nr and PFAM annotation results were imported into Blast2GO program, a software package that retrieves GO terms, allowing gene functions to be determined and compared [57].

In order to gain an overview of gene pathways networks, KEGG pathways were assigned to the unigenes using the online KEGG Automatic Annotation Server, (http://www.genome.jp/kegg/kaas/). The bi-directional best hit (BBH) method was used for KEGG Orthology (KO) assignment [58]. The output of KEGG analysis here includes KO assignments and KEGG pathways populated with the KO assignments.

### DGE data analysis

#### Reads mapping to the reference genome

Transcriptome data were selected as the reference. Reads were aligned to the reference genome using RSEM (v1.2.0) software package [59].

#### Quantification of gene expression level and differential expression analysis

HTSeq software (www-huber.embl.de/users/anders/HTSeq/) was used to count the reads mapped to each orthologous genomic region. For all comparisons, read counts were normalized to the aligned RPKM [60] to obtain the relative levels of expression. RPKM > 0.3 was defined as the threshold of significant gene expression. Differential expression analysis was performed using the R packages of DESeq for comparisons among samples with two biological replicates [61]. The correlation of the detected number of counts between parallel libraries was assessed statistically by calculating the Pearson correlation. *P*-values (adjusted for false discovery rate) were generated for each gene in pair-wise comparisons among samples. The *P*-values were adjusted using the method described by Benjamini and Hochberg [62]. A corrected *P*-value of 0.05 was set as the threshold for significant differential expression.

Variance-stabilized data obtained using DESeq was used to generate the heatmaps of differentially expressed genes. Clustering analysis was performed using the command hclust in R, and the heatmap was drown using the R packages of ggplot2 and pheatmap.

#### Gene ontology (GO) and KEGG enrichment analysis of differentially expressed genes

GO enrichment analysis of differentially expressed genes was implemented using the GOseq R package, in which gene length bias was corrected. GO terms with corrected *P*-values below 0.05 were considered significantly enriched by differentially expressed genes.

KEGG is a database resource meant to facilitate understanding of the high-level functions and utilities of biological systems, such as the cells, organisms, and ecosystems, using molecular-level information, especially large-scale molecular datasets generated by genome sequencing and other high-throughput experimental technologies (http://www.genome.jp/kegg/). KOBAS software was here used to test the statistical enrichment of differentially expressed genes in KEGG pathways. Pathways with corrected *P*-values below than 0.05 were considered significantly enriched by differentially expressed genes.

### Quantitative real-time PCR (qRT-PCR) validation

Total RNA was extracted as described for DGE library preparation and sequencing. For the first-strand cDNA synthesis experiment, a Transcriptor First Stand cDNA Synthesis Kit (Roche) was used following the manufacturer’s instructions. Quantitative real-time PCR was conducted using SYBR Green dye (LightCycle® 480 SYBR Green I Master). The selected genes were verified using the LightCycle® 480 Real-Time PCR System with the following cycling conditions: 95°C for 5min, followed by 45 cycles of 95°C for 10 s, 60°C for 10 s and 72°C for 20s. Specificity of the qPCR product was analyzed by melting curve analysis. The sequences of the primers used are given in Additional file 12. β-actin (*ACT3*) and translation initiation factor 4A (*eIF4a*) served as internal controls. The 2^-△△Ct^ method was used to calculate relative gene expression values [63].

### Availability of supporting data

The data sets supporting the results of this article are available in the Sequence Read Archive (SRA), accessible through NCBI BioProject ID PRJNA235353 for the transcriptome data (https://www.ncbi.nlm.nih.gov/sra/?term=PRJNA235353) and PRJNA236059 for the DGE data (https://www.ncbi.nlm.nih.gov/sra/?term=PRJNA236059).

## Additional files

Additional file 1: Table S1. KEGG enrichment of the 8 subclusters). Additional file 2: Table S2. The top 100 up-regulated unigenes (annotated) in CS compared with NT. Additional file 3: Table S3. The top 100 down-regulated unigenes (annotated) in CS compared with NT. Additional file 4: Table S4. GO enrichment analysis of (down and up)-regulated genes in CS compared with NT. Additional file 5: Table S5. The top 100 up-regulated unigenes (annotated) in FS compared with NT. Additional file 6: Table S6. The top 100 down-regulated unigenes (annotated) in FS compared with NT. Additional file 7: Table S7. GO enrichment analysis of (down an up)-regulated genes in FS compared with NT. Additional file 8: Table S8. The top 100 up-regulated unigenes (annotated) in HT compared with NT. Additional file 9: Table S9. The top 100 down-regulated unigenes (annotated) in HT compared with NT. Additional file 10: Table S10. GO enrichment analysis of (down and up)-regulated genes in HT compared with NT. Additional file 11: Table S11. Expression abundance of HSP genes in the eight samples. Additional file 12: Table S12. Primers used in qRT-PCR for validating differentially expressed genes.
