# Supplementary material for: Comparative transcriptome profiling of Pyropia yezoensis (Ueda) M.S. Hwang & H.G. Choi in response to temperature stresses
Source: BMC Genomics. 2015 Jun 17;16(1):463. doi: 10.1186/s12864-015-1586-1 (PMC4470342; doi:10.1186/s12864-015-1586-1)
Supplement: Additional file 1: Table S1. — KEGG enrichment of the 8 subclusters). [file 12864_2015_1586_MOESM1_ESM.docx]

Table S1 KEGG enrichment of the 8 subclusters

| subcluster1 | |
| --- | --- |
| Ribosome [PATH:ko03010] | comp13408_c0,comp13209_c0,comp6823_c0,comp13507_c0,comp13089_c0,comp7124_c0,comp7209_c0,comp7073_c0,comp58370_c0,comp12074_c0,comp10909_c0,comp7232_c0,comp11227_c0,comp13324_c0,comp6025_c0,comp13138_c0,comp11239_c0,comp12838_c0,comp2748_c0,comp10789_c0,comp7102_c0,comp11957_c0,comp11202_c0,comp6732_c0,comp10545_c0,comp10406_c0,comp11150_c0,comp9511_c0,comp2819_c0,comp10581_c0,comp8806_c0,comp8097_c0,comp6431_c0,comp10163_c1,comp10868_c0,comp14412_c0,comp13890_c0,comp6573_c0,comp7000_c0,comp27562_c0,comp7095_c0,comp13873_c0,comp10801_c0,comp13139_c0,comp2755_c0,comp14696_c0,comp155526_c0,comp7206_c0,comp9454_c0,comp11105_c0,comp3713_c0,comp13389_c0,comp6441_c0,comp6841_c0,comp13275_c0,comp10124_c0,comp11230_c0,comp11581_c0,comp17713_c0,comp6504_c0,comp6926_c0,comp13133_c0,comp11967_c0,comp2747_c0,comp10002_c0,comp12759_c0,comp8581_c0,comp13033_c0,comp10420_c0,comp10020_c0,comp9575_c0,comp6466_c0, |
| RNA transport [PATH:ko03013] | comp11671_c0,comp10575_c0,comp12177_c0,comp12266_c0,comp12843_c0,comp9801_c0,comp11000_c0,comp12651_c0,comp10286_c0,comp7063_c0,comp10570_c0,comp12534_c0,comp12713_c0,comp6480_c0,comp12828_c0,comp9151_c0,comp9822_c0,comp8579_c0,comp114500_c0,comp2909_c0,comp41216_c0,comp12375_c0,comp11992_c0,comp190927_c0,comp12312_c0, |
| subcluster2 | |
| Glycerolipid metabolism [PATH:ko00561] | comp11240_c0,comp12081_c0,comp9785_c0,comp8982_c0, |
| Fructose and mannose metabolism [PATH:ko00051] | comp11190_c0,comp6763_c0,comp10612_c0, |
| Pentose phosphate pathway [PATH:ko00030] | comp11190_c0,comp6763_c0,comp10612_c0, |
| Glutathione metabolism [PATH:ko00480] | comp41051_c0,comp33242_c0,comp9305_c0, |
| Carbon fixation in photosynthetic organisms [PATH:ko00710] | comp11190_c0,comp6763_c0,comp10612_c0, |
| Glycolysis / Gluconeogenesis [PATH:ko00010] | comp11190_c0,comp6763_c0,comp10612_c0, |
| subcluster3 | |
| Pentose phosphate pathway [PATH:ko00030] | comp6553_c0,comp2776_c0,comp9869_c0,comp13417_c0,comp9917_c0, |
| Starch and sucrose metabolism [PATH:ko00500] | comp6553_c0,comp12509_c0,comp12745_c0,comp11920_c0, |
| Pyruvate metabolism [PATH:ko00620] | comp10803_c0,comp5070_c0,comp10093_c0,comp8622_c0,comp7107_c0, |
| ABC transporters [PATH:ko02010] | comp69373_c0,comp12898_c0,comp3024_c0,comp88136_c0,comp99642_c0, |
| Carbon fixation pathways in prokaryotes [PATH:ko00720] | comp10803_c0,comp5070_c0,comp10093_c0,comp8622_c0, |
| Glycine, serine and threonine metabolism [PATH:ko00260] | comp12773_c0,comp11241_c0,comp7107_c0, |
| Carbon metabolism [PATH:ko01200] | comp10803_c0,comp12773_c0,comp13146_c0,comp5070_c0,comp9209_c0,comp10093_c0,comp2776_c0,comp9869_c0,comp11241_c0,comp8622_c0,comp9700_c0,comp13417_c0,comp9917_c0, |
| Glycolysis / Gluconeogenesis [PATH:ko00010] | comp6553_c0,comp13146_c0,comp9869_c0,comp13417_c0, |
| Steroid biosynthesis [PATH:ko00100] | comp12415_c0,comp50541_c0,comp3187_c0, |
| Alanine, aspartate and glutamate metabolism [PATH:ko00250] | comp10088_c0,comp6411_c0,comp12368_c0, |
| Glyoxylate and dicarboxylate metabolism [PATH:ko00630] | comp6411_c0,comp11241_c0,comp8741_c0,comp12368_c0,comp7107_c0, |
| Biosynthesis of amino acids [PATH:ko01230] | comp10088_c0,comp13146_c0,comp45722_c0,comp2776_c0,comp6411_c0,comp11241_c0,comp12368_c0,comp9917_c0, |
| Carbon fixation in photosynthetic organisms [PATH:ko00710] | comp10803_c0,comp13146_c0,comp5070_c0,comp9209_c0,comp10093_c0,comp2776_c0,comp9869_c0,comp8622_c0,comp9700_c0,comp13417_c0,comp9917_c0, |
| Arginine and proline metabolism [PATH:ko00330] | comp10088_c0,comp45722_c0,comp6411_c0,comp12368_c0, |
| subcluster4 | |
| Ribosome biogenesis in eukaryotes [PATH:ko03008] | comp8873_c0,comp6767_c0,comp12722_c0,comp12750_c0,comp164630_c0,comp10883_c0,comp12413_c0,comp1825_c0,comp12463_c0,comp38338_c0,comp21758_c0,comp107947_c0,comp11468_c0,comp63056_c0, |
| mRNA surveillance pathway [PATH:ko03015] | comp1850_c0,comp7947_c0,comp12768_c0,comp11495_c0,comp12803_c0,comp8445_c0,comp1512_c0,comp9148_c0,comp1490_c0,comp11319_c0, |
| PI3K-Akt signaling pathway [PATH:ko04151] | comp22866_c0,comp3904_c0,comp9303_c0,comp12768_c0,comp12342_c0,comp12803_c0,comp12949_c0,comp10120_c0,comp12381_c0,comp12618_c0, |
| Circadian rhythm - plant [PATH:ko04712] | comp12750_c0,comp10883_c0,comp11471_c0, |
| Regulation of autophagy [PATH:ko04140] | comp9303_c0,comp12949_c0,comp12618_c0, |
| RNA transport [PATH:ko03013] | comp7947_c0,comp12413_c0,comp11250_c0,comp8445_c0,comp76372_c0,comp9148_c0,comp1490_c0,comp2721_c0,comp11319_c0, |
| Inositol phosphate metabolism [PATH:ko00562] | comp2370_c0,comp8159_c0,comp12399_c0,comp11940_c0, |
| Circadian rhythm [PATH:ko04710] | comp9303_c0,comp12949_c0,comp12618_c0, |
| Wnt signaling pathway [PATH:ko04310] | comp10958_c0,comp12750_c0,comp10883_c0,comp12690_c0, |
| subcluster5 | |
| Pyrimidine metabolism [PATH:ko00240] | comp80069_c0,comp711_c0,comp3180_c0,comp33723_c0,comp47216_c0,comp6718_c0,comp5064_c0,comp65494_c0,comp61265_c0,comp126100_c0,comp12148_c0,comp3522_c0,comp125875_c0,comp71123_c0,comp64359_c0,comp67061_c0, |
| Ribosome biogenesis in eukaryotes [PATH:ko03008] | comp1816_c0,comp86663_c0,comp580_c0,comp11810_c0,comp10458_c0,comp11523_c0,comp4685_c0,comp12252_c0,comp9512_c0,comp1445_c0,comp24859_c0,comp94161_c0, |
| Alanine, aspartate and glutamate metabolism [PATH:ko00250] | comp10267_c0,comp85769_c0,comp3180_c0,comp33723_c0,comp6718_c0,comp5064_c0,comp1052_c0,comp61265_c0,comp126100_c0,comp12148_c0,comp64359_c0, |
| RNA polymerase [PATH:ko03020] | comp80069_c0,comp711_c0,comp47216_c0,comp65494_c0,comp3522_c0,comp125875_c0,comp71123_c0,comp67061_c0, |
| MAPK signaling pathway [PATH:ko04010] | comp9552_c0,comp11281_c0,comp12206_c0,comp9265_c0,comp10884_c0,comp11345_c0, |
| mTOR signaling pathway [PATH:ko04150] | comp12206_c0,comp24303_c0,comp9265_c0,comp10781_c0,comp10245_c0,comp3294_c0,comp97049_c0, |
| Cysteine and methionine metabolism [PATH:ko00270] | comp2756_c0,comp11221_c0,comp8755_c0,comp3101_c0,comp12001_c0,comp6880_c0,comp11856_c0, |
| One carbon pool by folate [PATH:ko00670] | comp11221_c0,comp8755_c0,comp12001_c0,comp11856_c0, |
| Selenocompound metabolism [PATH:ko00450] | comp11221_c0,comp8755_c0,comp12001_c0,comp11856_c0, |
| PI3K-Akt signaling pathway [PATH:ko04151] | comp24303_c0,comp9265_c0,comp10781_c0,comp10245_c0,comp11345_c0,comp3294_c0,comp97049_c0, |
| Pentose phosphate pathway [PATH:ko00030] | comp12051_c0,comp12962_c0,comp8663_c0,comp12628_c0,comp12628_c1, |
| Purine metabolism [PATH:ko00230] | comp80069_c0,comp711_c0,comp12669_c0,comp47216_c0,comp1052_c0,comp65494_c0,comp3522_c0,comp125875_c0,comp71123_c0,comp67061_c0, |
| Spliceosome [PATH:ko03040] | comp11215_c0,comp11281_c0,comp41500_c0,comp11523_c0,comp629_c0,comp100994_c0,comp118403_c0, |
| Rap1 signaling pathway [PATH:ko04015] | comp2713_c0,comp9265_c0,comp11345_c0, |
| Regulation of actin cytoskeleton [PATH:ko04810] | comp2713_c0,comp9265_c0,comp11345_c0, |
| Biosynthesis of amino acids [PATH:ko01230] | comp12669_c0,comp11221_c0,comp8755_c0,comp3101_c0,comp12001_c0,comp85769_c0,comp9502_c0,comp11856_c0,comp11176_c0,comp12051_c0,comp8663_c0, |
| Starch and sucrose metabolism [PATH:ko00500] | comp6379_c0,comp12628_c0,comp12628_c1, |
| subcluster6 | |
| Mismatch repair [PATH:ko03430] | comp146088_c0,comp118100_c0,comp12045_c0,comp3888_c0,comp9728_c0,comp8909_c0,comp27108_c0,comp709_c0,comp7355_c0,comp8100_c0,comp54925_c0,comp10479_c0, |
| Lysine biosynthesis [PATH:ko00300] | comp47938_c0,comp12738_c0,comp11089_c0,comp7737_c0,comp2113_c0,comp12392_c0, |
| DNA replication [PATH:ko03030] | comp146088_c0,comp118100_c0,comp12324_c0,comp12045_c0,comp3888_c0,comp8909_c0,comp27108_c0,comp11306_c0,comp7355_c0,comp8100_c0,comp54925_c0,comp10479_c0, |
| Pantothenate and CoA biosynthesis [PATH:ko00770] | comp9393_c0,comp10339_c0,comp9867_c0,comp4086_c0,comp12277_c0, |
| Porphyrin and chlorophyll metabolism [PATH:ko00860] | comp6061_c0,comp12723_c0,comp127485_c0,comp6949_c0,comp6665_c0,comp2099_c0,comp10647_c0,comp10154_c0,comp12331_c0,comp10101_c0,comp8717_c0, |
| Glutathione metabolism [PATH:ko00480] | comp6286_c0,comp8331_c0,comp6562_c0,comp12813_c0,comp9904_c0,comp11350_c0,comp6406_c0,comp12439_c0,comp174580_c0,comp6830_c0,comp9414_c0,comp12616_c0, |
| Valine, leucine and isoleucine biosynthesis [PATH:ko00290] | comp9393_c0,comp10339_c0,comp9867_c0,comp3759_c0,comp4086_c0,comp12277_c0, |
| Metabolism of xenobiotics by cytochrome P450 [PATH:ko00980] | comp8331_c0,comp9945_c0,comp12439_c0,comp9945_c1, |
| Base excision repair [PATH:ko03410] | comp11461_c0,comp8909_c0,comp1643_c0,comp11306_c0,comp7355_c0,comp40533_c0, |
| Biosynthesis of amino acids [PATH:ko01230] | comp47938_c0,comp12738_c0,comp11089_c0,comp7737_c0,comp10539_c0,comp9393_c0,comp12944_c0,comp4044_c0,comp11728_c0,comp10339_c0,comp2113_c0,comp9867_c0,comp12392_c0,comp61575_c0,comp3759_c0,comp4086_c0,comp11191_c0,comp12277_c0,comp12548_c0,comp6959_c0,comp4172_c0,comp6454_c0,comp8049_c0,comp8653_c0,comp93105_c0, |
| Arginine and proline metabolism [PATH:ko00330] | comp11340_c0,comp12738_c0,comp8545_c0,comp12371_c0,comp4044_c0,comp11728_c0,comp61575_c0,comp12548_c0,comp6959_c0,comp8049_c0, |
| Plant-pathogen interaction [PATH:ko04626] | comp12130_c0,comp3278_c0,comp1207_c0,comp11005_c0, |
| Protein export [PATH:ko03060] | comp2752_c0,comp81411_c0,comp9814_c0,comp11336_c0,comp113039_c0,comp40823_c0, |
| Photosynthesis - antenna proteins [PATH:ko00196] | comp13022_c0,comp2743_c0,comp11278_c0,comp12671_c0, |
| Cysteine and methionine metabolism [PATH:ko00270] | comp47938_c0,comp8545_c0,comp11089_c0,comp7737_c0,comp12944_c0,comp2113_c0,comp12392_c0,comp1717_c0,comp6959_c0, |
| Nucleotide excision repair [PATH:ko03420] | comp146088_c0,comp118100_c0,comp12045_c0,comp3888_c0,comp8909_c0,comp27108_c0,comp11306_c0,comp7355_c0,comp54925_c0, |
| Glycerolipid metabolism [PATH:ko00561] | comp11974_c0,comp8570_c0,comp12026_c0,comp1207_c0,comp11005_c0, |
| subcluster7 | |
| Protein processing in endoplasmic reticulum [PATH:ko04141] | comp6360_c0,comp2735_c0,comp2731_c0,comp2728_c0,comp8302_c0,comp10216_c0,comp12181_c1, |
| subcluster8 | |
| Photosynthesis - antenna proteins [PATH:ko00196] | comp11928_c0,comp6762_c0,comp11049_c0,comp11965_c0, |
| Peroxisome [PATH:ko04146] | comp10372_c0,comp7430_c0,comp12668_c0,comp3626_c0, |
| cAMP signaling pathway [PATH:ko04024] | comp10703_c0,comp56579_c0,comp3791_c0,comp12668_c0,comp29533_c0, |
| Protein processing in endoplasmic reticulum [PATH:ko04141] | comp12208_c0,comp2719_c0,comp6544_c0,comp7076_c0,comp11269_c0,comp12915_c0, |
| Ubiquitin mediated proteolysis [PATH:ko04120] | comp12208_c0,comp2719_c0,comp10335_c0,comp9053_c0, |
| Fatty acid metabolism [PATH:ko01212] | comp11937_c0,comp12668_c0,comp704_c0, |
