# Supplementary material for: Comparative transcriptome profiling of Pyropia yezoensis (Ueda) M.S. Hwang & H.G. Choi in response to temperature stresses
Source: BMC Genomics. 2015 Jun 17;16(1):463. doi: 10.1186/s12864-015-1586-1 (PMC4470342; doi:10.1186/s12864-015-1586-1)
Supplement: Additional file 2: Table S2. — The top 100 up-regulated unigenes (annotated) in CS compared with NT. [file 12864_2015_1586_MOESM2_ESM.docx]

Table S2 The top 100 up-regulated unigenes (annotated) in CS compared with NT

| gene_id | log_2_(CS/NT) | Gene Length | Description |
| --- | --- | --- | --- |
| comp1593_c0 | 3.9949 | 264 | ferredoxin [Arthrospira maxima CS-328] |
| comp5533_c0 | 3.3608 | 291 | Adenylate cyclase associated (CAP) N terminal |
| comp95989_c0 | 3.331 | 212 | Adenylate cyclase associated (CAP) N terminal |
| comp66341_c0 | 3.3048 | 543 | 60s Acidic ribosomal protein |
| comp11877_c0 | 3.0747 | 457 | Structural protein 2 |
| comp12536_c0 | 3.0406 | 2150 | predicted protein [Ostreococcus lucimarinus CCE9901] |
| comp29599_c0 | 3.0191 | 526 | Alphaherpesvirus tegument protein US9 |
| comp6945_c0 | 2.9848 | 535 | high light inducible protein [Pyropia yezoensis] |
| comp38004_c0 | 2.9709 | 258 | Homeobox KN domain |
| comp11131_c0 | 2.905 | 747 | high light inducible protein [Pyropia yezoensis] |
| comp5589_c0 | 2.8928 | 360 | Porphyra yezoensis high light inducible protein (Hli) mRNA, complete cds |
| comp145110_c0 | 2.8688 | 237 | ATPase family associated with various cellular activities (AAA) |
| comp8873_c0 | 2.7823 | 389 | mmr1/hsr1 GTP binding protein, putative [Pediculus humanus corporis] |
| comp12531_c0 | 2.7428 | 842 | hypothetical protein SORBIDRAFT_04g004620 [Sorghum bicolor] |
| comp1235_c0 | 2.715 | 632 | Mediator complex subunit 3 fungal//DM DNA binding domain//Ilarvirus coat protein |
| comp3460_c0 | 2.6733 | 463 | Potassium channel Kv1.4 tandem inactivation domain//Anti-sigma-K factor rskA |
| comp25919_c0 | 2.6057 | 1031 | hypothetical protein PTSG_09650 [Salpingoeca sp. ATCC 50818] |
| comp103820_c0 | 2.5986 | 219 | Potassium channel Kv1.4 tandem inactivation domain |
| comp7746_c0 | 2.5596 | 337 | Gammaherpesvirus capsid protein |
| comp5605_c0 | 2.4901 | 279 | DUF547-domain-containing protein [Coccomyxa subellipsoidea C-169] |
| comp11916_c0 | 2.4495 | 1804 | delta-9 fatty acid desaturase [Cyanidioschyzon merolae] |
| comp12653_c1 | 2.4478 | 535 | DEAD/DEAH box helicase family protein [Oxytricha trifallax] |
| comp10275_c0 | 2.4477 | 1489 | hypothetical protein AURANDRAFT_28650, partial [Aureococcus anophagefferens] |
| comp5390_c0 | 2.4459 | 261 | Plant self-incompatibility response (SCRL) protein |
| comp3268_c0 | 2.4427 | 535 | UV radiation resistance protein and autophagy-related subunit 14//Vesiculovirus phosphoprotein |
| comp9435_c0 | 2.4106 | 892 | Actin regulatory protein (Wiskott-Aldrich syndrome protein) |
| comp9045_c0 | 2.4086 | 338 | hypothetical protein MICPUCDRAFT_55736 [Micromonas pusilla CCMP1545] |
| comp318534_c0 | 2.4009 | 205 | unnamed protein product [Blastocystis hominis] |
| comp3062_c0 | 2.3882 | 673 | Cold shock protein DEAD box A//PAS fold |
| comp9165_c0 | 2.3641 | 1496 | F-actin binding |
| comp10066_c0 | 2.3557 | 1008 | conserved hypothetical protein [Phytophthora infestans T30-4] |
| comp1945_c0 | 2.3518 | 262 | cadherin-like protein, partial [Polyplectropus hovmoelleri] |
| comp12653_c0 | 2.3458 | 1171 | hypothetical protein SELMODRAFT_107027 [Selaginella moellendorffii] |
| comp92873_c0 | 2.343 | 269 | hypothetical protein SELMODRAFT_123861 [Selaginella moellendorffii] |
| comp11372_c0 | 2.3377 | 1389 | PREDICTED: hypothetical protein LOC100698576 [Oreochromis niloticus] |
| comp11348_c0 | 2.332 | 1422 | Collagens (type IV and type XIII), and related proteins |
| comp10752_c0 | 2.3011 | 202 | REV protein (anti-repression trans-activator protein)//FhuF 2Fe-2S C-terminal domain |
| comp93088_c0 | 2.2939 | 285 | EMG1/NEP1 methyltransferase |
| comp211969_c0 | 2.2912 | 273 | methyltransferase, FkbM family [Oscillatoriales cyanobacterium JSC-12] |
| comp410_c0 | 2.2666 | 333 | Serine/threonine protein kinase |
| comp3679_c0 | 2.2533 | 585 | Serine/threonine protein kinase |
| comp9658_c0 | 2.2378 | 487 | insulysin [Nannochloropsis gaditana CCMP526] |
| comp3933_c0 | 2.2299 | 202 | Transcription factor TFIIB repeat |
| comp42059_c0 | 2.2176 | 1066 | CCAAT/enhancer-binding protein zeta-like protein, partial [Equus caballus] |
| comp42149_c0 | 2.2167 | 813 | hypothetical protein VOLCADRAFT_40547 [Volvox carteri f. nagariensis] |
| comp88136_c0 | 2.2048 | 236 | PREDICTED: similar to multidrug resistance associated protein 2, partial [Hydra magnipapillata] |
| comp105606_c0 | 2.2037 | 272 | Putative caatt-binding transcription factor/60s ribosomal subunit bioproteinsis protein, partial [Rhipicephalus pulchellus] |
| comp12429_c0 | 2.2031 | 2420 | nitrate transporter [Pyropia yezoensis] |
| comp104281_c0 | 2.1974 | 510 | haloacid dehalogenase-like hydrolase [Chlamydomonas reinhardtii] |
| comp6937_c0 | 2.196 | 591 | predicted protein [Nematostella vectensis] |
| comp138834_c0 | 2.1885 | 386 | Ribosomal L39 protein |
| comp26687_c0 | 2.1768 | 355 | predicted protein [Hordeum vulgare subsp. vulgare] |
| comp9224_c0 | 2.1672 | 1033 | predicted protein [Physcomitrella patens subsp. patens] |
| comp126100_c0 | 2.1618 | 232 | hypothetical protein ACD_41C00058G0001, partial [uncultured bacterium] |
| comp9507_c0 | 2.1544 | 715 | Brf1-like TBP-binding domain//Sodium ion transport-associated |
| comp12349_c0 | 2.1242 | 764 | unnamed protein product [Blastocystis hominis] |
| comp67563_c0 | 2.1178 | 820 | predicted protein [Nematostella vectensis] |
| comp86410_c0 | 2.094 | 278 | MatE |
| comp57753_c0 | 2.0704 | 219 | Centromere protein H (CENP-H)//Intermediate filament head (DNA binding) region |
| comp6674_c0 | 2.066 | 605 | hypothetical protein CHLNCDRAFT_19166 [Chlorella variabilis] |
| comp10237_c0 | 2.0566 | 203 | NADH-ubiquinone oxidoreductase-G iron-sulfur binding region |
| comp28037_c0 | 2.0188 | 677 | putative signal transduction protein [Meiothermus ruber DSM 1279] |
| comp12244_c0 | 2.0025 | 697 | hypothetical protein RCJMB04_18j18 [Gallus gallus] |
| comp8494_c0 | 1.9975 | 561 | Putative trna and rrna cytosine-c5-methylase nucleolar protein nol1/nop2 [Ixodes ricinus] |
| comp68634_c0 | 1.9965 | 225 | Serine/threonine protein kinase |
| comp65162_c0 | 1.9958 | 583 | hypothetical protein GTHECHR2145 [Guillardia theta] |
| comp38921_c0 | 1.9896 | 411 | hypothetical protein UM02262.1 [Ustilago maydis 521] |
| comp10350_c0 | 1.9875 | 1015 | Potato type II proteinase inhibitor family//4Fe-4S binding domain//GAF domain//4Fe-4S binding domain |
| comp11861_c0 | 1.9789 | 1232 | delta-6 fatty acid desaturase [Mortierella alpina] |
| comp10631_c0 | 1.9755 | 1254 | heat shock protein Hsp20 [Mesoflavibacter zeaxanthinifaciens S86] |
| comp1032_c0 | 1.9725 | 660 | hypothetical protein DFA_08722 [Dictyostelium fasciculatum] |
| comp10168_c0 | 1.9716 | 511 | Domain of unknown function (DUF3474) |
| comp6703_c0 | 1.9649 | 982 | Fatty acid desaturase |
| comp12360_c0 | 1.9557 | 1151 | hypothetical protein Rleg8DRAFT_5503 [Rhizobium leguminosarum bv. trifolii WU95] |
| comp85533_c0 | 1.9529 | 562 | PREDICTED: LOW QUALITY PROTEIN: ornithine decarboxylase [Vitis vinifera] |
| comp6340_c0 | 1.9432 | 376 | Ribosomal protein S30//Nuclear envelope localisation domain |
| comp9892_c0 | 1.9419 | 464 | hypothetical protein [Marinobacter adhaerens HP15] |
| comp21853_c0 | 1.9343 | 476 | group IV decarboxylase [Dictyostelium fasciculatum] |
| comp33723_c0 | 1.9296 | 940 | aspartate carbamoyltransferase [Cryptococcus gattii WM276] |
| comp9512_c0 | 1.9175 | 774 | PREDICTED: U3 small nucleolar RNA-associated protein 15 homolog [Ovis aries] |
| comp77348_c0 | 1.9146 | 368 | ornithine decarboxylase [Auricularia delicata TFB-10046 SS5] |
| comp64522_c0 | 1.9054 | 509 | CG-1 domain |
| comp10421_c0 | 1.8969 | 911 | hypothetical protein BRAFLDRAFT_125653 [Branchiostoma floridae] |
| comp8242_c0 | 1.8969 | 812 | hypothetical protein CAOG_00072 [Capsaspora owczarzaki ATCC 30864] |
| comp9070_c0 | 1.8895 | 824 | hypothetical protein AURANDRAFT_34008 [Aureococcus anophagefferens] |
| comp34050_c0 | 1.8871 | 393 | Transcription factor IIA, alpha/beta subunit//ARC105 or Med15 subunit of Mediator complex non-fungal |
| comp66595_c0 | 1.8851 | 236 | zinc finger protein [Schistosoma mansoni] |
| comp1445_c0 | 1.8792 | 836 | hypothetical protein RO3G_16413 [Rhizopus oryzae RA 99-880] |
| comp5716_c0 | 1.8732 | 315 | Major intrinsic protein |
| comp69373_c0 | 1.8711 | 561 | ATP-binding cassette transporter YOR1 [Coprinopsis cinerea okayama7#130] |
| comp28190_c0 | 1.8677 | 378 | Syndecan domain//Peptidase family S49 N-terminal//DDHD domain//Translocation protein Sec62//Reticulon |
| comp50179_c0 | 1.8663 | 210 | Coronavirus nucleocapsid protein |
| comp11420_c0 | 1.865 | 1449 | hypothetical protein GUITHDRAFT_122270 [Guillardia theta CCMP2712] |
| comp12458_c0 | 1.8526 | 1390 | Mediator complex subunit 3 fungal//Ferric reductase NAD binding domain |
| comp105032_c0 | 1.8378 | 364 | Serine/threonine-protein kinase 6 [Acromyrmex echinatior] |
| comp107156_c0 | 1.8375 | 306 | beta-galactosidase [Granulicella tundricola MP5ACTX9] |
| comp12731_c0 | 1.819 | 1195 | cytochrome P450 [Rhizobium leguminosarum bv. viciae USDA 2370] |
| comp73939_c0 | 1.817 | 800 | protein MAK16 |
| comp9327_c0 | 1.8122 | 533 | WASP-interacting protein VRP1/WIP, contains WH2 domain |
| comp8636_c0 | 1.8092 | 734 | hypothetical protein DFA_11779 [Dictyostelium fasciculatum] |
