# Supplementary material for: Comparative transcriptome profiling of Pyropia yezoensis (Ueda) M.S. Hwang & H.G. Choi in response to temperature stresses
Source: BMC Genomics. 2015 Jun 17;16(1):463. doi: 10.1186/s12864-015-1586-1 (PMC4470342; doi:10.1186/s12864-015-1586-1)
Supplement: Additional file 3: Table S3. — The top 100 down-regulated unigenes (annotated) in CS compared with NT. [file 12864_2015_1586_MOESM3_ESM.docx]

Table S3 The top 100 down-regulated unigenes (annotated) in CS compared with NT

| gene_id | log_2_(CS/NT) | Gene Length | Description |
| --- | --- | --- | --- |
| comp135345_c0 | -5.0518 | 277 | hypothetical protein GUITHDRAFT_162841, partial [Guillardia theta CCMP2712] |
| comp146614_c0 | -4.5751 | 290 | Protein of unknown function (DUF3628) |
| comp54612_c0 | -4.3746 | 444 | hypothetical protein [Tuber melanosporum Mel28] |
| comp32053_c0 | -4.0847 | 255 | principal sigma factor [Pleurocapsa sp. PCC 7327] |
| comp71037_c0 | -4.0608 | 368 | RING-variant domain |
| comp36345_c0 | -3.8361 | 233 | Collagens (type IV and type XIII), and related proteins |
| comp81881_c0 | -3.6785 | 270 | Methylmalonyl-CoA mutase |
| comp54582_c0 | -3.6645 | 223 | PAP_fibrillin//Adenylate kinase, active site lid//Rubredoxin |
| comp2904_c0 | -3.6075 | 353 | RNA polymerase sigma factor rpoD [Microcystis aeruginosa TAIHU98] |
| comp2726_c0 | -3.4497 | 694 | Citrate transporter//Hepatitis C virus non-structural protein NS4a |
| comp21552_c0 | -3.3392 | 495 | Oxygen evolving enhancer protein 3 (PsbQ) |
| comp16520_c0 | -3.3373 | 501 | Mpv17 / PMP22 family |
| comp3261_c0 | -3.3355 | 367 | NADH pyrophosphatase zinc ribbon domain |
| comp11424_c0 | -3.2509 | 811 | Bacterial regulatory protein, arsR family |
| comp8344_c0 | -3.2058 | 336 | Serine/threonine protein kinase |
| comp12969_c0 | -3.1351 | 2664 | Uncharacterized WD repeat-containing protein C2A9.03 OS=Schizosaccharomyces pombe (strain 972 / ATCC 24843) GN=SPBC2A9.03 PE=2 SV=2 |
| comp57269_c0 | -3.0895 | 371 | Actin regulatory protein (Wiskott-Aldrich syndrome protein) |
| comp58055_c0 | -3.0519 | 294 | hypothetical protein GUITHDRAFT_121359 [Guillardia theta CCMP2712] |
| comp45715_c0 | -2.9144 | 282 | Huwentoxin-II family |
| comp59011_c0 | -2.8911 | 276 | hypothetical protein NGA_0726700 [Nannochloropsis gaditana CCMP526] |
| comp8549_c0 | -2.8696 | 1174 | Ycf46 [Pyropia yezoensis] |
| comp12871_c0 | -2.8483 | 1984 | Alpha-galactosidase [Paenibacillus lactis 154] |
| comp3414_c0 | -2.6459 | 761 | BTK motif |
| comp50826_c0 | -2.55 | 261 | Bromovirus coat protein |
| comp9723_c0 | -2.4232 | 552 | Atrial natriuretic peptide//Mediator of RNA pol II transcription subunit 19 |
| comp9058_c0 | -2.4054 | 268 | FOG: RRM domain |
| comp54824_c0 | -2.3822 | 573 | predicted protein [Bathycoccus prasinos] |
| comp5144_c0 | -2.365 | 464 | Cutinase//Phospholipase/Carboxylesterase//Dienelactone hydrolase family//alpha/beta hydrolase fold//Alpha/Beta hydrolase family of unknown function (DUF1234) |
| comp10223_c0 | -2.3568 | 336 | Proteolipid membrane potential modulator |
| comp1730_c0 | -2.2908 | 453 | Major Facilitator Superfamily |
| comp10933_c0 | -2.2817 | 586 | Methyltransferase small domain |
| comp6892_c0 | -2.2773 | 285 | Protein of unknown function (DUF3628) |
| comp6475_c0 | -2.2552 | 1360 | FAD binding domain of DNA photolyase//DNA photolyase |
| comp6620_c0 | -2.2522 | 437 | putative methyltransferase [Pontibacter sp. BAB1700] |
| comp1717_c0 | -2.238 | 295 | 3-mercaptopyruvate sulfurtransferase [Azospirillum brasilense Sp245] |
| comp2787_c0 | -2.216 | 519 | hypothetical protein GUITHDRAFT_135885 [Guillardia theta CCMP2712] |
| comp8062_c0 | -2.1992 | 478 | conserved hypothetical protein [Toxoplasma gondii GT1] |
| comp11618_c0 | -2.1883 | 860 | hypothetical protein CY0110_10617 [Cyanothece sp. CCY0110] |
| comp11355_c0 | -2.1675 | 1109 | methyltransferase [Fibrella aestuarina BUZ 2] |
| comp102134_c0 | -2.1279 | 215 | hypothetical protein GUITHDRAFT_98988 [Guillardia theta CCMP2712] |
| comp6542_c0 | -2.1027 | 387 | Bowman-Birk serine protease inhibitor family |
| comp6753_c0 | -2.088 | 521 | hypothetical protein CHLNCDRAFT_137065 [Chlorella variabilis] |
| comp40545_c0 | -2.082 | 273 | Ankyrin repeat |
| comp4510_c0 | -2.0603 | 373 | Serine/threonine protein kinase |
| comp7768_c0 | -2.0508 | 730 | high-affinity Fe2+/Pb2+ permease [Chamaesiphon minutus PCC 6605] |
| comp2572_c0 | -2.0068 | 547 | Ribosomal protein L19e |
| comp7019_c0 | -1.9718 | 338 | Hepatitis core antigen |
| comp9725_c0 | -1.9599 | 1131 | Leucine carboxyl methyltransferase |
| comp5598_c0 | -1.9569 | 346 | predicted protein [Physcomitrella patens subsp. patens] |
| comp679_c0 | -1.9466 | 351 | DTHCT (NUC029) region |
| comp1171_c0 | -1.9463 | 353 | Rho GTPase effector BNI1 and related formins |
| comp3539_c0 | -1.9457 | 727 | hypothetical protein [Salinibacter ruber DSM 13855] |
| comp8827_c0 | -1.9447 | 501 | Nuclear pore complex, Nup98 component (sc Nup145/Nup100/Nup116) |
| comp61069_c0 | -1.9423 | 215 | Ribosomal protein L4/L1 family |
| comp12608_c1 | -1.9378 | 3605 | trehalose-6-phosohate synthase [Monostroma angicava] |
| comp10279_c0 | -1.9251 | 1168 | hypothetical protein HMPREF9625_00080 [Oribacterium sp. ACB1] |
| comp79224_c0 | -1.9195 | 437 | ArgJ family//GATA zinc finger |
| comp9724_c0 | -1.9019 | 492 | Uroporphyrinogen-III synthase HemD |
| comp11608_c0 | -1.9016 | 1391 | hypothetical protein SORBIDRAFT_09g019180 [Sorghum bicolor] |
| comp9425_c0 | -1.8987 | 531 | Vesicle coat complex COPII, subunit SEC24/subunit SFB2 |
| comp10827_c0 | -1.8971 | 1758 | Car6 protein, partial [Mus musculus] |
| comp55892_c0 | -1.8947 | 369 | DNA-directed DNA polymerase III PolC [Oscillatoria acuminata PCC 6304] |
| comp7549_c0 | -1.8839 | 654 | protein kinase domain containing protein [Acanthamoeba castellanii str. Neff] |
| comp7018_c0 | -1.8777 | 292 | Serine/threonine protein kinase |
| comp63937_c0 | -1.8438 | 355 | ABC transporter-like protein [Cyanothece sp. PCC 7822] |
| comp2671_c0 | -1.8358 | 367 | Collagens (type IV and type XIII), and related proteins |
| comp11545_c0 | -1.8224 | 739 | hypothetical protein CHLREDRAFT_167070 [Chlamydomonas reinhardtii] |
| comp12323_c0 | -1.8072 | 1283 | hypothetical protein GUITHDRAFT_95599 [Guillardia theta CCMP2712] |
| comp11089_c0 | -1.8059 | 1057 | hypothetical protein VITISV_034964 [Vitis vinifera] |
| comp8508_c0 | -1.8053 | 744 | hypothetical protein [Synechococcus elongatus PCC 6301] |
| comp6704_c0 | -1.804 | 263 | Porphyra yezoensis putative plastid 1-deoxy-D-xylulose 5-phosphate synthase precursor (DXS) mRNA, partial cds; nuclear gene for plastid product |
| comp11598_c0 | -1.7994 | 504 | photosystem II extrinsic protein [Galdieria sulphuraria] |
| comp61282_c0 | -1.7917 | 265 | Paired amphipathic helix repeat |
| comp9027_c0 | -1.7877 | 1531 | PREDICTED: cyclin-P3-1-like [Glycine max] |
| comp1565_c0 | -1.7865 | 228 | Ubiquinol-cytochrome c reductase 8 kDa, N-terminal |
| comp12693_c0 | -1.7833 | 3782 | predicted protein [Phaeodactylum tricornutum CCAP 1055/1] |
| comp11462_c0 | -1.7819 | 1040 | Glutathione S-transferase-like protein [Cyanothece sp. CCY0110] |
| comp8483_c0 | -1.7672 | 507 | ankyrin repeat-containing protein [Bacillus megaterium WSH-002] |
| comp3001_c0 | -1.7632 | 495 | taurine-transporting ATPase [Calothrix sp. PCC 7507] |
| comp8983_c0 | -1.7597 | 951 | Insulin-like growth factor binding protein |
| comp21161_c0 | -1.7596 | 432 | Carbonic anhydrase [Cronobacter turicensis 564] |
| comp3956_c0 | -1.7596 | 402 | Aminoglycoside/hydroxyurea antibiotic resistance kinase |
| comp6020_c0 | -1.7582 | 370 | PREDICTED: UPF0420 protein C16orf58 homolog [Glycine max] |
| comp1184_c0 | -1.7479 | 698 | hypothetical protein [Pseudoalteromonas atlantica T6c] |
| comp9867_c0 | -1.7313 | 667 | dihydroxyacid dehydratase/phosphogluconate dehydratase [Moorea producta 3L] |
| comp13128_c0 | -1.7287 | 825 | Actin regulatory protein (Wiskott-Aldrich syndrome protein) |
| comp9407_c0 | -1.727 | 482 | CASK-interacting adaptor protein (caskin) and related proteins with ankyrin repeats and SAM domain |
| comp33553_c0 | -1.7267 | 277 | conserved unknown protein [Ectocarpus siliculosus] |
| comp40652_c0 | -1.6908 | 464 | Sigma-70, non-essential region//Procyclic acidic repetitive protein (PARP)//CDC45-like protein |
| comp6339_c0 | -1.6885 | 883 | Peroxidase//Raf-like Ras-binding domain |
| comp36970_c0 | -1.6861 | 298 | Betaherpesvirus UL82/83 protein N terminus |
| comp10944_c0 | -1.6791 | 931 | predicted protein [Phaeodactylum tricornutum CCAP 1055/1] |
| comp10463_c0 | -1.6772 | 529 | predicted protein [Thalassiosira pseudonana CCMP1335] |
| comp58474_c0 | -1.6601 | 309 | Orbivirus helicase VP6 |
| comp103543_c0 | -1.6562 | 417 | Peptide methionine sulfoxide reductase |
| comp11452_c0 | -1.6542 | 529 | Actin regulatory protein (Wiskott-Aldrich syndrome protein) |
| comp95778_c0 | -1.6522 | 314 | F-box associated region |
| comp6734_c0 | -1.651 | 968 | hypothetical protein GUITHDRAFT_160183, partial [Guillardia theta CCMP2712] |
| comp12923_c0 | -1.6458 | 2192 | hypothetical protein TcasGA2_TC003960 [Tribolium castaneum] |
| comp12108_c0 | -1.6381 | 714 | Rhodanese-related sulfurtransferase [Cupriavidus sp. HMR-1] |
