# Supplementary material for: Comparative transcriptome profiling of Pyropia yezoensis (Ueda) M.S. Hwang & H.G. Choi in response to temperature stresses
Source: BMC Genomics. 2015 Jun 17;16(1):463. doi: 10.1186/s12864-015-1586-1 (PMC4470342; doi:10.1186/s12864-015-1586-1)
Supplement: Additional file 4: Table S4. — GO enrichment analysis of (down and up)-regulated genes in CS compared with NT. [file 12864_2015_1586_MOESM4_ESM.docx]

Table S4 GO enrichment analysis of (down/up)-regulated genes in CS compared with NT

GO enrichment analysis of the down-regulated genes in CS compared with NT

| GO_accession | Description | Corrected_pValue | DEG_item | Bg_item |
| --- | --- | --- | --- | --- |
| **biological_process** | | | | |
| GO:0006418 | tRNA aminoacylation for protein translation | 0.000103 | 17 | 93 |
| GO:0043038 | amino acid activation | 0.000103 | 17 | 94 |
| GO:0043039 | tRNA aminoacylation | 0.000103 | 17 | 94 |
| GO:0006399 | tRNA metabolic process | 0.009432 | 20 | 171 |
| **molecular_function** |  |  |  |  |
| GO:0004812 | aminoacyl-tRNA ligase activity | 0.000103 | 17 | 93 |
| GO:0016875 | ligase activity, forming carbon-oxygen bonds | 0.000103 | 17 | 93 |
| GO:0016876 | ligase activity, forming aminoacyl-tRNA and related compounds | 0.000103 | 17 | 93 |

GO enrichment analysis of the up-regulated genes in CS compared with NT

| GO_accession | Description | Corrected_pValue | DEG_item | Bg_item |
| --- | --- | --- | --- | --- |
| **molecular_function** | | | | |
| GO:0016717 | oxidoreductase activity, acting on paired donors, with oxidation of a pair of donors resulting in the reduction of molecular oxygen to two molecules of water | 0.000413 | 6 | 6 |

DEG_item means the number of DEGs in related to this GO function.

Bg_item means the number of all genes in this GO function.
