# Supplementary material for: Comparative transcriptome profiling of Pyropia yezoensis (Ueda) M.S. Hwang & H.G. Choi in response to temperature stresses
Source: BMC Genomics. 2015 Jun 17;16(1):463. doi: 10.1186/s12864-015-1586-1 (PMC4470342; doi:10.1186/s12864-015-1586-1)
Supplement: Additional file 5: Table S5. — The top 100 up-regulated unigenes (annotated) in FS compared with NT. [file 12864_2015_1586_MOESM5_ESM.docx]

Table S5 The top 100 up-regulated unigenes (annotated) in FS compared with NT

| gene_id | log_2_(FS/NT) | Gene Length | Description |
| --- | --- | --- | --- |
| comp314577_c0 | 7.0725 | 222 | ABC-1 domain-containing protein [Fischerella sp. JSC-11] |
| comp42149_c0 | 6.646 | 813 | hypothetical protein VOLCADRAFT_40547 [Volvox carteri f. nagariensis] |
| comp241213_c0 | 6.303 | 252 | hydrolase activity, acting on ester bonds |
| comp12349_c0 | 5.8452 | 764 | unnamed protein product [Blastocystis hominis] |
| comp95989_c0 | 5.7869 | 212 | Adenylate cyclase associated (CAP) N terminal |
| comp9099_c0 | 5.7562 | 475 | Phlyctochytrium planicorne isolate AFTOL-ID 628 28S ribosomal RNA gene, partial sequence |
| comp725_c0 | 5.7532 | 364 | Protein of unknown function DUF1838 [Ktedonobacter racemifer DSM 44963] |
| comp224284_c0 | 5.7103 | 256 | Ribosomal L39 protein |
| comp10142_c0 | 5.542 | 318 | Actin regulatory protein (Wiskott-Aldrich syndrome protein) |
| comp11877_c0 | 5.0775 | 457 | Structural protein 2 |
| comp7746_c0 | 4.9343 | 337 | Gammaherpesvirus capsid protein |
| comp11889_c0 | 4.9007 | 1000 | His-Cys box protein [Porphyra umbilicalis] |
| comp38004_c0 | 4.8128 | 258 | Homeobox KN domain |
| comp100986_c0 | 4.734 | 367 | Collagens (type IV and type XIII), and related proteins |
| comp10407_c0 | 4.6868 | 419 | U1 small nuclear ribonucleoprotein (RRM superfamily) |
| comp6703_c0 | 4.4698 | 982 | Fatty acid desaturase |
| comp6768_c0 | 4.4268 | 536 | Porphyra miniata voucher GWS002155 large subunit ribosomal RNA gene, partial sequence |
| comp12536_c0 | 4.3473 | 2150 | predicted protein [Ostreococcus lucimarinus CCE9901] |
| comp142313_c0 | 4.2944 | 283 | RIP metalloprotease RseP [Gloeocapsa sp. PCC 73106] |
| comp10168_c0 | 4.236 | 511 | Domain of unknown function (DUF3474) |
| comp138834_c0 | 4.1998 | 386 | Ribosomal L39 protein |
| comp1593_c0 | 4.1318 | 264 | ferredoxin [Arthrospira maxima CS-328] |
| comp12494_c0 | 4.0895 | 773 | thioredoxin reductase [Fischerella sp. JSC-11] |
| comp64414_c0 | 3.9331 | 249 | Hok/gef family |
| comp9475_c0 | 3.9197 | 370 | elogin binding protein-like protein [Griffithsia japonica] |
| comp9045_c0 | 3.729 | 338 | hypothetical protein MICPUCDRAFT_55736 [Micromonas pusilla CCMP1545] |
| comp10349_c0 | 3.7193 | 907 | thioredoxin-disulfide reductase [Oscillatoria acuminata PCC 6304] |
| comp410_c0 | 3.7097 | 333 | Serine/threonine protein kinase |
| comp163611_c0 | 3.6837 | 244 | Aminotransferase class IV |
| comp12531_c0 | 3.671 | 842 | hypothetical protein SORBIDRAFT_04g004620 [Sorghum bicolor] |
| comp227912_c0 | 3.6661 | 241 | Porphyra yezoensis PyACT1 gene for actin, partial cds |
| comp104281_c0 | 3.6503 | 510 | haloacid dehalogenase-like hydrolase [Chlamydomonas reinhardtii] |
| comp163132_c0 | 3.6387 | 214 | conserved hypothetical protein [Brevundimonas sp. BAL3] |
| comp10092_c0 | 3.6374 | 813 | DnaJ central domain |
| comp3460_c0 | 3.6244 | 463 | Potassium channel Kv1.4 tandem inactivation domain//Anti-sigma-K factor rskA |
| comp11131_c0 | 3.5678 | 747 | high light inducible protein [Pyropia yezoensis] |
| comp82056_c0 | 3.5176 | 216 | sugar phosphate phosphatase [Salmonella enterica subsp. enterica serovar Enteritidis str. SE30663] |
| comp11372_c0 | 3.4804 | 1389 | PREDICTED: hypothetical protein LOC100698576 [Oreochromis niloticus] |
| comp9165_c0 | 3.4717 | 1496 | F-actin binding |
| comp79318_c0 | 3.4343 | 387 | WD domain, G-beta repeat |
| comp65162_c0 | 3.4203 | 583 | hypothetical protein GTHECHR2145 [Guillardia theta] |
| comp5390_c0 | 3.4114 | 261 | Plant self-incompatibility response (SCRL) protein |
| comp12360_c0 | 3.3413 | 1151 | hypothetical protein Rleg8DRAFT_5503 [Rhizobium leguminosarum bv. trifolii WU95] |
| comp41454_c0 | 3.3228 | 317 | Serine/threonine protein kinase |
| comp9070_c0 | 3.3022 | 824 | hypothetical protein AURANDRAFT_34008 [Aureococcus anophagefferens] |
| comp3933_c0 | 3.2906 | 202 | Transcription factor TFIIB repeat |
| comp3578_c0 | 3.2753 | 425 | hypothetical protein OsI_17367 [Oryza sativa Indica Group] |
| comp3679_c0 | 3.2633 | 585 | Serine/threonine protein kinase |
| comp70985_c0 | 3.2428 | 471 | Deoxyribodipyrimidine photo-lyase (DNA photolyase) (Photoreactivating enzyme) [Bradyrhizobium sp. ORS 285] |
| comp12150_c0 | 3.1846 | 1095 | Actin regulatory protein (Wiskott-Aldrich syndrome protein) |
| comp8199_c0 | 3.1613 | 543 | Phytochrome region |
| comp175270_c0 | 3.153 | 211 | Antenna complex alpha/beta subunit |
| comp1235_c0 | 3.1527 | 632 | Mediator complex subunit 3 fungal//DM DNA binding domain//Ilarvirus coat protein |
| comp11916_c0 | 3.1365 | 1804 | delta-9 fatty acid desaturase [Cyanidioschyzon merolae] |
| comp27643_c0 | 3.1299 | 367 | Serine/threonine protein kinase |
| comp399_c0 | 3.1269 | 384 | Cytosol aminopeptidase family, N-terminal domain |
| comp12081_c0 | 3.0713 | 890 | hypothetical protein GUITHDRAFT_115024 [Guillardia theta CCMP2712] |
| comp113482_c0 | 3.053 | 244 | high light inducible protein [Pyropia yezoensis] |
| comp3242_c0 | 3.0461 | 484 | WASP-interacting protein VRP1/WIP, contains WH2 domain |
| comp19466_c0 | 3.0214 | 224 | NADH-ubiquinone oxidoreductase B18 subunit (NDUFB7) |
| comp1362_c0 | 3.0212 | 290 | hypothetical protein Lepto7375DRAFT_6574 [Leptolyngbya sp. PCC 7375] |
| comp13032_c0 | 3.0127 | 895 | PGR5 [Pyropia yezoensis] |
| comp25919_c0 | 2.9985 | 1031 | hypothetical protein PTSG_09650 [Salpingoeca sp. ATCC 50818] |
| comp10633_c0 | 2.9559 | 642 | Protein conjugation factor involved in autophagy |
| comp160072_c0 | 2.9149 | 233 | Gammaherpesvirus capsid protein//Mycobacterium 19 kDa lipoprotein antigen//Suppressor of forked protein (Suf) |
| comp11764_c0 | 2.9102 | 1004 | Potato leaf roll virus readthrough protein |
| comp74596_c0 | 2.8164 | 306 | predicted protein [Physcomitrella patens subsp. patens] |
| comp28037_c0 | 2.7989 | 677 | putative signal transduction protein [Meiothermus ruber DSM 1279] |
| comp167012_c0 | 2.7862 | 216 | Wiskott Aldrich syndrome proteins |
| comp11001_c0 | 2.767 | 551 | High potential iron-sulfur protein |
| comp61629_c0 | 2.7663 | 321 | UV radiation resistance protein and autophagy-related subunit 14//Fusaric acid resistance protein family//Fez1 |
| comp5589_c0 | 2.7599 | 360 | Porphyra yezoensis high light inducible protein (Hli) mRNA, complete cds |
| comp7043_c0 | 2.7543 | 497 | Ribonucleases P/MRP protein subunit POP1 |
| comp9785_c0 | 2.7409 | 539 | galactolipid galactosyltransferase [Chlamydomonas reinhardtii] |
| comp7659_c0 | 2.7393 | 679 | deoxyribodipyrimidine photolyase family [Verrucomicrobiae bacterium DG1235] |
| comp8982_c0 | 2.7357 | 312 | digalactosyldiacylglycerol synthase [Chromera velia] |
| comp1029_c0 | 2.7209 | 425 | protein of unknown function DUF399 [Ectocarpus siliculosus] |
| comp12215_c0 | 2.7192 | 1525 | Prenyltransferase and squalene oxidase repeat |
| comp8559_c0 | 2.7157 | 509 | Fibrillins and related proteins containing Ca2+-binding EGF-like domains |
| comp112434_c0 | 2.6921 | 241 | Gammaherpesvirus capsid protein//Myb-like DNA-binding domain |
| comp9327_c0 | 2.6877 | 533 | WASP-interacting protein VRP1/WIP, contains WH2 domain |
| comp28190_c0 | 2.6748 | 378 | Syndecan domain//Peptidase family S49 N-terminal//DDHD domain//Translocation protein Sec62//Reticulon |
| comp10752_c0 | 2.6472 | 202 | REV protein (anti-repression trans-activator protein)//FhuF 2Fe-2S C-terminal domain |
| comp55688_c0 | 2.6434 | 254 | Keratin, high-sulphur matrix protein//Metallothionein |
| comp11900_c0 | 2.6114 | 1099 | 2-methyl-6-phytylbenzoquinone methyltranferase [Hevea brasiliensis] |
| comp35328_c0 | 2.6017 | 757 | glycoside hydrolase [Coraliomargarita akajimensis DSM 45221] |
| comp11861_c0 | 2.5825 | 1232 | delta-6 fatty acid desaturase [Mortierella alpina] |
| comp6945_c0 | 2.577 | 535 | high light inducible protein [Pyropia yezoensis] |
| comp880_c0 | 2.5692 | 435 | predicted protein [Ostreococcus lucimarinus CCE9901] |
| comp10807_c0 | 2.5668 | 822 | predicted protein [Thalassiosira pseudonana CCMP1335] |
| comp21196_c0 | 2.53 | 341 | conserved unknown protein [Ectocarpus siliculosus] |
| comp38118_c0 | 2.5245 | 269 | Ankyrin repeat |
| comp28606_c0 | 2.5174 | 300 | hypothetical protein Mic7113_1105 [Microcoleus sp. PCC 7113] |
| comp6636_c0 | 2.5114 | 990 | Gastrula zinc finger protein XlCGF26.1 [Lepeophtheirus salmonis] |
| comp46176_c0 | 2.5101 | 818 | Otx1 transcription factor |
| comp101828_c0 | 2.4999 | 327 | hypothetical protein THAOC_37874 [Thalassiosira oceanica] |
| comp8636_c0 | 2.4396 | 734 | hypothetical protein DFA_11779 [Dictyostelium fasciculatum] |
| comp107851_c0 | 2.4339 | 243 | CLIP, MHC2 interacting//ATP synthase B/B' CF(0)//Guanylate-binding protein, C-terminal domain//Flagellar protein FliT |
| comp11485_c0 | 2.4186 | 750 | hypothetical protein GUITHDRAFT_74279 [Guillardia theta CCMP2712] |
| comp6340_c0 | 2.4147 | 376 | Ribosomal protein S30//Nuclear envelope localisation domain |
