# Supplementary material for: Comparative transcriptome profiling of Pyropia yezoensis (Ueda) M.S. Hwang & H.G. Choi in response to temperature stresses
Source: BMC Genomics. 2015 Jun 17;16(1):463. doi: 10.1186/s12864-015-1586-1 (PMC4470342; doi:10.1186/s12864-015-1586-1)
Supplement: Additional file 6: Table S6. — The top 100 down-regulated unigenes (annotated) in FS compared with NT. [file 12864_2015_1586_MOESM6_ESM.docx]

Table S6 The top 100 down-regulated unigenes (annotated) in FS compared with NT

| gene_id | log_2_(FS/NT) | Gene Length | Description |
| --- | --- | --- | --- |
| comp11545_c0 | -6.4742 | 739 | hypothetical protein CHLREDRAFT_167070 [Chlamydomonas reinhardtii] |
| comp6542_c0 | -6.2704 | 387 | Bowman-Birk serine protease inhibitor family |
| comp7549_c0 | -6.1648 | 654 | protein kinase domain containing protein [Acanthamoeba castellanii str. Neff] |
| comp10661_c0 | -5.6842 | 453 | Protein kinase domain containing protein [Tetrahymena thermophila] |
| comp602_c0 | -4.7867 | 551 | hypothetical protein FAES_3577 [Fibrella aestuarina BUZ 2] |
| comp9082_c0 | -4.5937 | 387 | hypothetical protein GUITHDRAFT_58085, partial [Guillardia theta CCMP2712] |
| comp12571_c1 | -4.1874 | 878 | Signal transduction protein Enabled, contains WH1 domain |
| comp3261_c0 | -4.1447 | 367 | NADH pyrophosphatase zinc ribbon domain |
| comp9083_c0 | -4.0518 | 606 | FOG: RRM domain |
| comp45715_c0 | -3.8776 | 282 | Huwentoxin-II family |
| comp7263_c0 | -3.8363 | 424 | hypothetical protein THAPSDRAFT_261650 [Thalassiosira pseudonana CCMP1335] |
| comp11280_c0 | -3.822 | 1236 | pyrophosphate--fructose-6-phosphate 1-phosphotransferase [Arabidopsis thaliana] |
| comp9224_c0 | -3.8155 | 1033 | predicted protein [Physcomitrella patens subsp. patens] |
| comp9435_c0 | -3.4559 | 892 | Actin regulatory protein (Wiskott-Aldrich syndrome protein) |
| comp7998_c0 | -3.4473 | 347 | Putative RNA polymerase II regulator |
| comp11348_c0 | -3.3543 | 1422 | Collagens (type IV and type XIII), and related proteins |
| comp12605_c0 | -3.2546 | 1208 | hypothetical protein BATDEDRAFT_36999 [Batrachochytrium dendrobatidis JAM81] |
| comp191011_c0 | -3.1868 | 258 | conserved unknown protein [Ectocarpus siliculosus] |
| comp11686_c0 | -3.0774 | 2069 | putative hexose phosphate translocator [Galdieria sulphuraria] |
| comp1336_c0 | -3.0327 | 687 | Na+/H+ antiporter [Pyropia yezoensis] |
| comp38037_c0 | -2.9099 | 786 | Na+/H+ antiporter [Pyropia yezoensis] |
| comp9867_c0 | -2.8593 | 667 | dihydroxyacid dehydratase/phosphogluconate dehydratase [Moorea producta 3L] |
| comp21552_c0 | -2.7755 | 495 | Oxygen evolving enhancer protein 3 (PsbQ) |
| comp9393_c0 | -2.7424 | 824 | dihydroxy-acid dehydratase [Synechococcus sp. JA-3-3Ab] |
| comp476_c0 | -2.7411 | 733 | hypothetical protein Pse7429DRAFT_1835 [Pseudanabaena biceps PCC 7429] |
| comp4086_c0 | -2.7155 | 346 | dihydroxy-acid dehydratase [Amycolatopsis mediterranei U32] |
| comp11322_c0 | -2.5175 | 1209 | diphosphate-fructose-6-phosphate 1-phosphotransferase [Acanthamoeba castellanii str. Neff] |
| comp11411_c0 | -2.5035 | 1390 | uhpC gene product [Parachlamydia acanthamoebae UV-7] |
| comp51792_c0 | -2.468 | 338 | cell division cycle protein 20 [Phytophthora infestans T30-4] |
| comp1730_c0 | -2.4286 | 453 | Major Facilitator Superfamily |
| comp10003_c0 | -2.4002 | 990 | Outer membrane protein (OmpH-like)//Domain of unknown function (DUF3552)//Glycosyltransferase family 9 (heptosyltransferase)//Survival motor neuron protein (SMN)//General secretion pathway protein M |
| comp64002_c0 | -2.3323 | 451 | hypothetical protein PHYSODRAFT_536256 [Phytophthora sojae] |
| comp38251_c0 | -2.3208 | 593 | hypothetical protein GUITHDRAFT_102558 [Guillardia theta CCMP2712] |
| comp2043_c0 | -2.2979 | 328 | Gammaherpesvirus capsid protein |
| comp13632_c0 | -2.2978 | 925 | hypothetical protein ZEAMMB73_713834 [Zea mays] |
| comp7125_c0 | -2.2272 | 1210 | 2-isopropylmalate synthase [Crinalium epipsammum PCC 9333] |
| comp11750_c0 | -2.2257 | 749 | 2-isopropylmalate synthase [Nannochloropsis gaditana CCMP526] |
| comp10827_c0 | -2.216 | 1758 | Car6 protein, partial [Mus musculus] |
| comp79224_c0 | -2.2146 | 437 | ArgJ family//GATA zinc finger |
| comp12032_c0 | -2.204 | 1775 | PREDICTED: seryl-tRNA synthetase [Vitis vinifera] |
| comp6657_c0 | -2.1911 | 601 | glucose-6-phosphate 1-dehydrogenase [Cyanidium caldarium] |
| comp11841_c0 | -2.163 | 1169 | Tryptophan--tRNA ligase [Ectocarpus siliculosus] |
| comp34003_c0 | -2.1528 | 580 | Immunoreceptor tyrosine-based activation motif |
| comp12988_c0 | -2.1336 | 726 | C2 domain |
| comp10288_c0 | -2.1217 | 426 | Heat shock protein 9/12 |
| comp11355_c0 | -2.0858 | 1109 | methyltransferase [Fibrella aestuarina BUZ 2] |
| comp95544_c0 | -2.0794 | 625 | hypothetical protein OtV6_115c [Ostreococcus tauri virus RT-2011] |
| comp8744_c0 | -2.0778 | 1057 | glucose-6-phosphate 1-dehydrogenase [Cyanidium caldarium] |
| comp103463_c0 | -2.063 | 235 | Dual specificity phosphatase, catalytic domain containing protein [Oxytricha trifallax] |
| comp12444_c0 | -2.0557 | 698 | glutamate-1-semialdehyde-2,1-aminomutase [Cyanobium sp. PCC 7001] |
| comp65031_c0 | -2.0466 | 317 | Sigma-70 factor, region 1.2 |
| comp38818_c0 | -2.0449 | 211 | D-3-phosphoglycerate dehydrogenase [Leptospira borgpetersenii serovar Hardjo-bovis L550] |
| comp6620_c0 | -2.0422 | 437 | putative methyltransferase [Pontibacter sp. BAB1700] |
| comp144395_c0 | -2.0311 | 262 | dehydroascorbate reductase [Sesamum indicum] |
| comp10771_c0 | -1.9972 | 684 | NADH:flavin oxidoreductase [Cyanothece sp. PCC 7424] |
| comp11831_c0 | -1.9908 | 689 | hypothetical protein ECKD2_16568, partial [Escherichia coli KD2] |
| comp5790_c0 | -1.974 | 297 | Merozoite surface protein 1 (MSP1) C-terminus |
| comp12438_c0 | -1.9096 | 1952 | predicted protein [Ostreococcus lucimarinus CCE9901] |
| comp9125_c0 | -1.9055 | 1824 | glutamate-1-semialdehyde 2,1-aminomutase, chloroplastic [Coccomyxa subellipsoidea C-169] |
| comp5003_c0 | -1.895 | 697 | IPT/TIG domain |
| comp22403_c0 | -1.8766 | 598 | hypothetical protein Pse7367_0498 [Pseudanabaena sp. PCC 7367] |
| comp56701_c0 | -1.8698 | 314 | related to positive effector protein GCN20 [Sporisorium reilianum SRZ2] |
| comp3956_c0 | -1.8342 | 402 | Aminoglycoside/hydroxyurea antibiotic resistance kinase |
| comp69295_c0 | -1.828 | 312 | Pc21g23060 [Penicillium chrysogenum Wisconsin 54-1255] |
| comp5708_c0 | -1.8123 | 434 | Hypothetical protein ACA1_192610 [Acanthamoeba castellanii str. Neff] |
| comp2786_c0 | -1.7994 | 1670 | putative plastid geranylgeranyl reductase precusor [Pyropia yezoensis] |
| comp7933_c0 | -1.7897 | 779 | CPN60 protein [Guillardia theta] |
| comp30324_c0 | -1.7724 | 808 | chaperonin Cpn60 [Rhodomonas salina] |
| comp6753_c0 | -1.7616 | 521 | hypothetical protein CHLNCDRAFT_137065 [Chlorella variabilis] |
| comp9765_c0 | -1.7613 | 486 | cyclin B [Chroomonas mesostigmatica CCMP1168] |
| comp8362_c0 | -1.7544 | 483 | ABC transporter C family protein [Dictyostelium fasciculatum] |
| comp12890_c0 | -1.7491 | 1535 | predicted protein [Physcomitrella patens subsp. patens] |
| comp9392_c0 | -1.7453 | 583 | NADH:flavin oxidoreductase, Old Yellow Enzyme family [Moorea producta 3L] |
| comp6992_c0 | -1.7442 | 1209 | Serine/threonine protein kinase |
| comp920_c0 | -1.7202 | 377 | Putative RNA polymerase II regulator |
| comp34193_c0 | -1.7177 | 717 | Membrane transport protein |
| comp12543_c0 | -1.7175 | 1415 | RNA polymerase II C-terminal domain-binding protein RA4, contains RPR and RRM domains |
| comp6751_c0 | -1.7012 | 635 | Mu DNA-binding domain |
| comp10339_c0 | -1.6947 | 1384 | branched chain amino acid aminotransferase [Ectocarpus siliculosus] |
| comp8876_c0 | -1.6906 | 751 | dhm exonuclease [Ectocarpus siliculosus] |
| comp6959_c0 | -1.6591 | 641 | aspartate aminotransferase, cytoplasmic, putative [Pediculus humanus corporis] |
| comp9285_c0 | -1.6579 | 1453 | hypothetical protein [Tuber melanosporum Mel28] |
| comp12263_c0 | -1.6527 | 2268 | hypothetical protein VOLCADRAFT_63305 [Volvox carteri f. nagariensis] |
| comp10456_c0 | -1.6521 | 424 | tryptophanyl-tRNA synthetase [Dictyostelium purpureum] |
| comp9366_c0 | -1.6455 | 474 | uncharacterized protein, partial [Phleum pratense] |
| comp9090_c0 | -1.6414 | 766 | Splicing coactivator SRm160/300, subunit SRm300 |
| comp3846_c0 | -1.6381 | 452 | SWI/SNF-related matrix-associated actin-dependent regulator of chromatin |
| comp7099_c0 | -1.6316 | 1338 | LysE type translocator |
| comp1184_c0 | -1.6241 | 698 | hypothetical protein [Pseudoalteromonas atlantica T6c] |
| comp43702_c0 | -1.6195 | 388 | D-3-phosphoglycerate dehydrogenase-like protein [Leishmania donovani] |
| comp47424_c0 | -1.6131 | 344 | Photosystem I reaction centre subunit N (PSAN or PSI-N) |
| comp8259_c0 | -1.6115 | 1174 | PREDICTED: aspartate aminotransferase, cytoplasmic-like [Brachypodium distachyon] |
| comp61779_c0 | -1.6025 | 303 | MscS family inner membrane protein ynaI [Waddlia chondrophila 2032/99] |
| comp12747_c0 | -1.6022 | 2279 | 3-deoxy-7-phosphoheptulonate synthase [Azospirillum brasilense Sp245] |
| comp12277_c0 | -1.5939 | 1702 | predicted protein [Micromonas sp. RCC299] |
| comp12185_c0 | -1.584 | 2114 | PREDICTED: similar to carboxylase:pyruvate/acetyl-coa/propionyl-coa [Ciona intestinalis] |
| comp11618_c0 | -1.5807 | 860 | hypothetical protein CY0110_10617 [Cyanothece sp. CCY0110] |
| comp6963_c0 | -1.5717 | 755 | hypothetical protein GTHECHR2161 [Guillardia theta] |
| comp7381_c0 | -1.57 | 887 | Glycosyl hydrolase family 3 C terminal domain//Methylamine dehydrogenase, L chain |
| comp10364_c0 | -1.5488 | 1282 | SWI-SNF chromatin-remodeling complex protein |
