# Supplementary material for: Comparative transcriptome profiling of Pyropia yezoensis (Ueda) M.S. Hwang & H.G. Choi in response to temperature stresses
Source: BMC Genomics. 2015 Jun 17;16(1):463. doi: 10.1186/s12864-015-1586-1 (PMC4470342; doi:10.1186/s12864-015-1586-1)
Supplement: Additional file 7: Table S7. — GO enrichment analysis of (down an up)-regulated genes in FS compared with NT. [file 12864_2015_1586_MOESM7_ESM.docx]

Table S7 GO enrichment analysis of (down/up)-regulated genes in FS compared with NT

GO enrichment analysis of the down-regulated genes in FS compared with NT

| GO_accession | Description | Corrected_pValue | DEG_item | Bg_item |
| --- | --- | --- | --- | --- |
| **biological_process** | | | | |
| GO:0006520 | cellular amino acid metabolic process | 0.002133 | 25 | 466 |
| GO:0019752 | carboxylic acid metabolic process | 0.002133 | 28 | 607 |
| GO:0043436 | oxoacid metabolic process | 0.002133 | 28 | 614 |
| GO:0006082 | organic acid metabolic process | 0.002133 | 28 | 616 |
| GO:0042180 | cellular ketone metabolic process | 0.005263 | 28 | 652 |
| GO:0009082 | branched-chain amino acid biosynthetic process | 0.034848 | 6 | 40 |
| GO:0008652 | cellular amino acid biosynthetic process | 0.039372 | 12 | 184 |
| GO:0042026 | protein refolding | 0.039372 | 3 | 6 |
| GO:0006007 | glucose catabolic process | 0.039372 | 8 | 71 |
| GO:0019320 | hexose catabolic process | 0.039372 | 8 | 71 |
| GO:0046365 | monosaccharide catabolic process | 0.039372 | 8 | 72 |
| GO:0009081 | branched-chain amino acid metabolic process | 0.040141 | 7 | 59 |
| GO:0016052 | carbohydrate catabolic process | 0.042344 | 8 | 78 |
| GO:0044724 | single-organism carbohydrate catabolic process | 0.042344 | 8 | 78 |
| GO:0044283 | small molecule biosynthetic process | 0.042344 | 17 | 359 |
| GO:0006006 | glucose metabolic process | 0.044507 | 8 | 79 |
| GO:0005996 | monosaccharide metabolic process | 0.044507 | 9 | 104 |
| GO:0044711 | single-organism biosynthetic process | 0.044507 | 17 | 382 |
| GO:0009110 | vitamin biosynthetic process | 0.044507 | 7 | 77 |
| GO:0042364 | water-soluble vitamin biosynthetic process | 0.044507 | 7 | 77 |
| GO:0006418 | tRNA aminoacylation for protein translation | 0.044507 | 8 | 93 |
| GO:0044281 | small molecule metabolic process | 0.044507 | 35 | 1119 |
| GO:0016053 | organic acid biosynthetic process | 0.044507 | 14 | 277 |
| GO:0046394 | carboxylic acid biosynthetic process | 0.044507 | 14 | 277 |
| GO:0043038 | amino acid activation | 0.044507 | 8 | 94 |
| GO:0043039 | tRNA aminoacylation | 0.044507 | 8 | 94 |

GO enrichment analysis of the up-regulated genes in FS compared with NT

| GO_accession | Description | Corrected_pValue | DEG_item | Bg_item |
| --- | --- | --- | --- | --- |
| **molecular_function** | | | | |
| GO:0016717 | oxidoreductase activity, acting on paired donors, with oxidation of a pair of donors resulting in the reduction of molecular oxygen to two molecules of water | 6.54E-07 | 6 | 6 |
| GO:0008964 | phosphoenolpyruvate carboxylase activity | 0.000444 | 4 | 4 |
| GO:0004611 | phosphoenolpyruvate carboxykinase activity | 0.001644 | 4 | 5 |

DEG_item means the number of DEGs in related to this GO function.

Bg_item means the number of all genes in this GO function.
