# Supplementary material for: Comparative transcriptome profiling of Pyropia yezoensis (Ueda) M.S. Hwang & H.G. Choi in response to temperature stresses
Source: BMC Genomics. 2015 Jun 17;16(1):463. doi: 10.1186/s12864-015-1586-1 (PMC4470342; doi:10.1186/s12864-015-1586-1)
Supplement: Additional file 8: Table S8. — The top 100 up-regulated unigenes (annotated) in HT compared with NT. [file 12864_2015_1586_MOESM8_ESM.docx]

Table S8 The top 100 up-regulated unigenes (annotated) in HT compared with NT

| gene_id | log_2_ (HT/NT) | Gene Length | Description |
| --- | --- | --- | --- |
| comp79409_c0 | 6.2811 | 342 | Kelch repeat and BTB domain-containing protein 10 [Salmo salar] |
| comp2723_c0 | 6.0353 | 749 | Hsp20/alpha crystallin family [Brevundimonas sp. BAL3] |
| comp73975_c0 | 5.8711 | 232 | Glutathione-dependent formaldehyde-activating enzyme |
| comp5570_c0 | 5.6343 | 395 | Elongation factor Tu GTP binding domain |
| comp193252_c0 | 4.7494 | 270 | IPT/TIG domain |
| comp649_c0 | 4.7177 | 444 | translation elongation factor G [Dictyoglomus thermophilum H-6-12] |
| comp3728_c0 | 4.5519 | 429 | translation elongation factor 2 (EF-2/EF-G) [Thermaerobacter subterraneus DSM 13965] |
| comp3228_c0 | 4.4963 | 471 | Lipoprotein Rz1 precursor |
| comp209552_c0 | 4.4066 | 305 | Halotolerance protein HAL3 (contains flavoprotein domain) |
| comp9596_c0 | 4.2311 | 583 | Collagens (type IV and type XIII), and related proteins |
| comp10631_c0 | 4.1114 | 1254 | heat shock protein Hsp20 [Mesoflavibacter zeaxanthinifaciens S86] |
| comp75876_c0 | 4.0724 | 369 | RecName: Full=R-phycoerythrin gamma chain, chloroplastic; Flags: Precursor |
| comp6703_c0 | 3.9661 | 982 | Fatty acid desaturase |
| comp11632_c0 | 3.9527 | 689 | PREDICTED: putative germin-like protein 2-1 [Vitis vinifera] |
| comp7838_c0 | 3.9018 | 736 | Galactoside-binding lectin |
| comp44647_c0 | 3.8906 | 686 | Hep_Hag |
| comp7914_c0 | 3.8816 | 568 | Collagens (type IV and type XIII), and related proteins |
| comp7679_c0 | 3.7496 | 213 | MMPL family |
| comp5612_c0 | 3.7271 | 274 | Ubiquitinol-cytochrome C reductase Fe-S subunit TAT signal |
| comp706_c0 | 3.7124 | 364 | hypothetical protein ACA1_367650 [Acanthamoeba castellanii str. Neff] |
| comp102063_c0 | 3.6661 | 398 | Orbivirus helicase VP6//Orexin receptor type 2 |
| comp10470_c0 | 3.6331 | 452 | Phospholipase/Carboxylesterase |
| comp6992_c0 | 3.6281 | 1209 | Serine/threonine protein kinase |
| comp6968_c0 | 3.5982 | 1070 | cupin-like protein [Ectocarpus siliculosus] |
| comp13016_c0 | 3.5792 | 809 | Metallo-beta-lactamase superfamily |
| comp17460_c0 | 3.5695 | 485 | Accessory gene regulator B |
| comp70778_c0 | 3.4982 | 509 | RNA pseudouridylate synthase |
| comp10292_c0 | 3.4974 | 373 | Actin regulatory protein (Wiskott-Aldrich syndrome protein) |
| comp86497_c0 | 3.4582 | 446 | Plasmodium ookinete surface protein Pvs28 |
| comp37549_c0 | 3.4545 | 481 | Cytochrome c oxidase subunit IV |
| comp12710_c0 | 3.362 | 2109 | hypothetical protein OsJ_01907 [Oryza sativa Japonica Group] |
| comp73913_c0 | 3.3591 | 672 | Neurohypophysial hormones, C-terminal Domain |
| comp9691_c0 | 3.32 | 945 | Universal stress protein YxiE OS=Bacillus subtilis (strain 168) GN=yxiE PE=3 SV=1 |
| comp115799_c0 | 3.3096 | 279 | Transcription regulator dachshund, contains SKI/SNO domain |
| comp3506_c0 | 3.2531 | 301 | Anti-sigma-K factor rskA |
| comp169153_c0 | 3.2499 | 330 | Lipoprotein Rz1 precursor |
| comp7776_c0 | 3.2331 | 641 | ZIP Zinc transporter family protein [Tetrahymena thermophila] |
| comp11661_c0 | 3.2126 | 989 | Lamprin |
| comp2772_c0 | 3.1762 | 1091 | Collagens (type IV and type XIII), and related proteins |
| comp230890_c0 | 3.1716 | 279 | Apo-citrate lyase phosphoribosyl-dephospho-CoA transferase |
| comp6644_c0 | 3.1626 | 714 | Anti-sigma-K factor rskA |
| comp5479_c0 | 3.1182 | 819 | NAD-dependent DNA ligase C4 zinc finger domain//Rubredoxin |
| comp127574_c0 | 3.116 | 289 | Cytochrome C oxidase copper chaperone (COX17) |
| comp2762_c0 | 3.1127 | 229 | Tc3 transposase |
| comp113737_c0 | 3.1112 | 225 | predicted protein [Populus trichocarpa] |
| comp6717_c0 | 3.0813 | 588 | Actin regulatory protein (Wiskott-Aldrich syndrome protein) |
| comp4205_c0 | 3.0735 | 278 | Anti-sigma-K factor rskA |
| comp1211_c0 | 3.0734 | 251 | DM DNA binding domain |
| comp36945_c0 | 3.0496 | 327 | InsA N-terminal domain |
| comp175315_c0 | 3.0372 | 227 | Nuclear transition protein 2 |
| comp8865_c0 | 3.0084 | 506 | Vesicle coat complex COPII, subunit SFB3 |
| comp9043_c0 | 2.9908 | 231 | carbonate dehydratase [Ochrobactrum anthropi CTS-325] |
| comp9006_c0 | 2.9577 | 948 | Iron-binding zinc finger CDGSH type |
| comp9617_c0 | 2.838 | 1285 | APC cysteine-rich region |
| comp11598_c0 | 2.8081 | 504 | photosystem II extrinsic protein [Galdieria sulphuraria] |
| comp51845_c0 | 2.748 | 461 | zinc permease family [Micromonas pusilla CCMP1545] |
| comp12992_c0 | 2.7445 | 1007 | Anaphylotoxin-like domain//UDP-glucoronosyl and UDP-glucosyl transferase//Magi 5 toxic peptide family |
| comp12753_c0 | 2.7423 | 1020 | conserved hypothetical protein [Phytophthora infestans T30-4] |
| comp11501_c0 | 2.7331 | 840 | ABC transporter [Salpingoeca sp. ATCC 50818] |
| comp169993_c0 | 2.7302 | 312 | Mitochondrial ATPase inhibitor, IATP//Tetrahydromethanopterin S-methyltransferase, subunit E |
| comp10917_c0 | 2.7227 | 2198 | hypothetical protein CHLNCDRAFT_134017 [Chlorella variabilis] |
| comp197760_c0 | 2.7171 | 370 | Ubiquitinol-cytochrome C reductase Fe-S subunit TAT signal |
| comp232815_c0 | 2.7017 | 288 | hypothetical protein FOXB_12793 [Fusarium oxysporum Fo5176] |
| comp13043_c0 | 2.6951 | 495 | Porphyra yezoensis polyubiquitin (PUBI-2) gene, complete cds |
| comp2785_c0 | 2.6944 | 1294 | hypothetical protein [Drosophila pseudoobscura] |
| comp602_c0 | 2.6873 | 551 | hypothetical protein FAES_3577 [Fibrella aestuarina BUZ 2] |
| comp1836_c0 | 2.674 | 544 | hypothetical protein BATDEDRAFT_8900 [Batrachochytrium dendrobatidis JAM81] |
| comp1716_c0 | 2.657 | 237 | InsA N-terminal domain |
| comp6579_c0 | 2.6507 | 1419 | hypothetical protein Cal7507_0697 [Calothrix sp. PCC 7507] |
| comp9515_c0 | 2.6503 | 276 | Selenoprotein S (SelS) |
| comp1866_c0 | 2.6357 | 611 | hypothetical protein VOLCADRAFT_108333 [Volvox carteri f. nagariensis] |
| comp8484_c0 | 2.5995 | 484 | BTK motif//Reticulon |
| comp29349_c0 | 2.5806 | 753 | Solute carrier family 35, member E3, putative [Acanthamoeba castellanii str. Neff] |
| comp97201_c0 | 2.5766 | 295 | Poly-beta-hydroxybutyrate polymerase (PhaC) N-terminus |
| comp86285_c0 | 2.5751 | 513 | Colipase, N-terminal domain |
| comp107156_c0 | 2.5741 | 306 | beta-galactosidase [Granulicella tundricola MP5ACTX9] |
| comp9035_c0 | 2.5517 | 751 | ASPO1527 [Pyropia yezoensis] |
| comp102991_c0 | 2.5504 | 368 | PREDICTED: dynein 8 kDa light chain, flagellar outer arm [Vitis vinifera] |
| comp196984_c0 | 2.5417 | 246 | ATP-dependent RNA helicase |
| comp103813_c0 | 2.5403 | 216 | Mediator complex subunit 3 fungal//Zinc knuckle |
| comp11889_c0 | 2.5115 | 1000 | His-Cys box protein [Porphyra umbilicalis] |
| comp157632_c0 | 2.5035 | 343 | Tetratricopeptide repeat |
| comp101832_c0 | 2.4954 | 309 | glycosyltransferase family 4 protein [Micromonas pusilla CCMP1545] |
| comp25906_c0 | 2.4895 | 221 | ASPO1527 [Pyropia yezoensis] |
| comp2337_c0 | 2.4741 | 731 | Collagens (type IV and type XIII), and related proteins |
| comp9302_c0 | 2.4705 | 1567 | WASP-interacting protein VRP1/WIP, contains WH2 domain |
| comp28577_c0 | 2.4626 | 590 | vanadium-dependent bromoperoxidase [Mesoflavibacter zeaxanthinifaciens S86] |
| comp12061_c0 | 2.4507 | 1140 | Nucleolar GTPase/ATPase p130 |
| comp10357_c0 | 2.4269 | 531 | U1 zinc finger |
| comp11672_c0 | 2.4258 | 1240 | ATPase, AAA domain containing protein [Acanthamoeba castellanii str. Neff] |
| comp398_c0 | 2.4178 | 573 | Mediator complex subunit 3 fungal |
| comp4494_c0 | 2.4171 | 325 | Fungal Zn(2)-Cys(6) binuclear cluster domain//Pyridoxine 5'-phosphate oxidase C-terminal dimerisation region |
| comp1995_c0 | 2.4162 | 254 | predicted protein [Thalassiosira pseudonana CCMP1335] |
| comp9774_c0 | 2.4082 | 318 | short chain dehydrogenase |
| comp3835_c0 | 2.3917 | 366 | 2,3-bisphosphoglycerate-independent phosphoglycerate mutase [Spirochaeta thermophila DSM 6192] |
| comp11186_c0 | 2.3896 | 837 | FK506-binding protein 1 [Ectocarpus siliculosus] |
| comp10216_c0 | 2.3691 | 514 | PREDICTED: protein disulfide-isomerase A6-like [Megachile rotundata] |
| comp47827_c0 | 2.3594 | 254 | Cytochrome oxidase c subunit VIb |
| comp10933_c0 | 2.3445 | 586 | Methyltransferase small domain |
| comp12858_c0 | 2.3334 | 1307 | beta-Ig-H3/fasciclin [Dinoroseobacter shibae DFL 12] |
