# Supplementary material for: Comparative transcriptome profiling of Pyropia yezoensis (Ueda) M.S. Hwang & H.G. Choi in response to temperature stresses
Source: BMC Genomics. 2015 Jun 17;16(1):463. doi: 10.1186/s12864-015-1586-1 (PMC4470342; doi:10.1186/s12864-015-1586-1)
Supplement: Additional file 9: Table S9. — The top 100 down-regulated unigenes (annotated) in HT compared with NT. [file 12864_2015_1586_MOESM9_ESM.docx]

Table S9 The top 100 down-regulated unigenes (annotated) in HT compared with NT

| gene_id | log_2_ (HT/NT) | Gene Length | Description |
| --- | --- | --- | --- |
| comp40248_c0 | -5.1168 | 524 | 3-oxoacyl-acyl-carrier-protein synthase 3 [Porphyra purpurea] |
| comp2518_c0 | -4.9744 | 519 | photosystem II 47 kDa protein [Pyropia yezoensis] |
| comp26794_c0 | -4.9636 | 459 | hypothetical chloroplast protein 38 [Pyropia yezoensis] |
| comp17713_c0 | -4.8076 | 395 | ribosomal protein L19 [Pyropia yezoensis] |
| comp3760_c1 | -4.6951 | 385 | ATP synthase CF0 B&apos; subunit [Pyropia yezoensis] |
| comp10163_c1 | -4.5217 | 836 | ribosomal protein S14 [Pyropia yezoensis] |
| comp73828_c0 | -4.3399 | 409 | photosystem II 47 kDa protein [Porphyra purpurea] |
| comp299944_c0 | -4.3306 | 235 | ribosomal protein L33 [Pyropia yezoensis] |
| comp23935_c0 | -4.2463 | 840 | photosystem II 47 kDa protein [Pyropia yezoensis] |
| comp2355_c0 | -4.1367 | 599 | photosystem I assembly protein Ycf4 [Pyropia yezoensis] |
| comp8712_c0 | -3.9887 | 1047 | RecName: Full=Ribulose bisphosphate carboxylase large chain; Short=RuBisCO large subunit |
| comp70838_c0 | -3.9711 | 283 | ATP synthase CF0 A subunit [Pyropia yezoensis] |
| comp2282_c0 | -3.8991 | 1051 | ribulose-1,5-bisphosphate carboxylase/oxygenase large subunit, partial (chloroplast) [Pyropia katadae] |
| comp4981_c0 | -3.8478 | 1030 | ATP synthase CF0 A subunit [Pyropia yezoensis] |
| comp30615_c0 | -3.7597 | 1029 | ATP synthase CF1 delta subunit [Pyropia yezoensis] |
| comp12501_c0 | -3.724 | 516 | RhoA GTPase effector DIA/Diaphanous |
| comp5144_c0 | -3.6614 | 464 | RhoA GTPase effector DIA/Diaphanous |
| comp53078_c0 | -3.6588 | 570 | photosystem I subunit XI [Porphyra purpurea] |
| comp6075_c0 | -3.5997 | 253 | magnesium-protoporphyrin IX monomethyl ester cyclase [Pyropia yezoensis] |
| comp42268_c0 | -3.5319 | 410 | GMP synthase [Thalassiosira pseudonana CCMP1335] |
| comp9482_c0 | -3.4819 | 658 | hypothetical protein VOLCADRAFT_100183 [Volvox carteri f. nagariensis] |
| comp9894_c1 | -3.4572 | 1412 | nitrate reductase [Gracilaria tenuistipitata] |
| comp11601_c0 | -3.4546 | 1573 | beta-Ig-H3/fasciclin [Dinoroseobacter shibae DFL 12] |
| comp58370_c0 | -3.4465 | 282 | ribosomal protein S18 [Pyropia yezoensis] |
| comp18288_c0 | -3.4081 | 310 | cytochrome b6/f complex subunit VIII [Porphyra purpurea] |
| comp158104_c0 | -3.3928 | 343 | GMP synthase [Thermincola potens JR] |
| comp58164_c0 | -3.3918 | 416 | nitrogen regulatory protein PII [Pyropia yezoensis] |
| comp12311_c0 | -3.3469 | 1771 | nitrate reductase [Gracilaria tenuistipitata] |
| comp85769_c0 | -3.2643 | 202 | synthetase I,carbamylphosphate |
| comp17041_c0 | -3.2376 | 353 | photosystem I subunit III [Pyropia yezoensis] |
| comp11664_c0 | -3.0831 | 479 | PAP2 superfamily |
| comp105397_c0 | -3.0572 | 204 | 6-O-methylguanine DNA methyltransferase, DNA binding domain |
| comp11700_c0 | -3.0209 | 807 | hypothetical protein TRIADDRAFT_64190 [Trichoplax adhaerens] |
| comp9373_c0 | -2.9905 | 240 | Predicted RNA-binding protein containing PIN domain and invovled in translation or RNA processing |
| comp8599_c0 | -2.9801 | 940 | ATP synthase CF1 alpha subunit [Pyropia yezoensis] |
| comp3679_c0 | -2.9787 | 585 | Serine/threonine protein kinase |
| comp111911_c0 | -2.9373 | 223 | hypothetical conserved protein [uncultured candidate division OP1 bacterium] |
| comp11534_c0 | -2.8993 | 1321 | hypothetical protein [Cryptosporidium hominis TU502] |
| comp12536_c0 | -2.8803 | 2150 | predicted protein [Ostreococcus lucimarinus CCE9901] |
| comp27562_c0 | -2.878 | 528 | ribosomal protein S4 [Pyropia yezoensis] |
| comp8400_c0 | -2.8691 | 462 | GMP synthase [Dichomitus squalens LYAD-421 SS1] |
| comp3272_c0 | -2.849 | 727 | hypothetical protein RB2501_08740 [Robiginitalea biformata HTCC2501] |
| comp4354_c0 | -2.8407 | 433 | hypothetical protein 114 [Pyropia yezoensis] |
| comp9435_c0 | -2.7909 | 892 | Actin regulatory protein (Wiskott-Aldrich syndrome protein) |
| comp15402_c0 | -2.7569 | 453 | photosystem I subunit III [Pyropia yezoensis] |
| comp12218_c0 | -2.7277 | 1269 | conserved unknown protein [Ectocarpus siliculosus] |
| comp10309_c0 | -2.705 | 718 | Mitochondrial ATPase inhibitor, IATP |
| comp9051_c0 | -2.7042 | 566 | hypothetical protein CHLNCDRAFT_144404 [Chlorella variabilis] |
| comp2756_c0 | -2.6998 | 893 | hypothetical protein VOLCADRAFT_74602 [Volvox carteri f. nagariensis] |
| comp11215_c0 | -2.6894 | 1972 | hypothetical protein BATDEDRAFT_27077 [Batrachochytrium dendrobatidis JAM81] |
| comp8127_c0 | -2.66 | 558 | hypothetical chloroplast protein 38 [Pyropia yezoensis] |
| comp11025_c0 | -2.6415 | 1112 | protein arginine N-methyltransferase [Chlamydomonas reinhardtii] |
| comp8741_c0 | -2.6369 | 1001 | phosphoglycolate phosphatase [Thalassiosira pseudonana CCMP1335] |
| comp12341_c0 | -2.6362 | 803 | Cwf15/Cwc15 cell cycle control protein//Sodium ion transport-associated//Transcription initiation factor IIF, alpha subunit (TFIIF-alpha)//Vesiculovirus matrix protein |
| comp6730_c0 | -2.6261 | 1229 | Poxvirus P21 membrane protein |
| comp60901_c0 | -2.6191 | 635 | photochlorophyllide reductase subunit B [Pyropia yezoensis] |
| comp12790_c0 | -2.6052 | 1852 | unnamed protein product [Blastocystis hominis] |
| comp5204_c0 | -2.6008 | 1305 | glycosyl transferase family 2 [Holophaga foetida DSM 6591] |
| comp10575_c0 | -2.5368 | 2274 | putative eukaryotic translation initiation factor 4A [Pyropia yezoensis] |
| comp11348_c0 | -2.5359 | 1422 | Collagens (type IV and type XIII), and related proteins |
| comp1888_c0 | -2.5201 | 294 | respiratory burst oxidase-like protein [Pyropia yezoensis] |
| comp9224_c0 | -2.5152 | 1033 | predicted protein [Physcomitrella patens subsp. patens] |
| comp6880_c0 | -2.5111 | 1061 | RecName: Full=Adenosylhomocysteinase; Short=AdoHcyase; AltName: Full=S-adenosyl-L-homocysteine hydrolase |
| comp5390_c0 | -2.494 | 261 | Plant self-incompatibility response (SCRL) protein |
| comp12284_c0 | -2.4838 | 1149 | Ectropic viral integration site 2A protein (EVI2A) |
| comp9788_c0 | -2.4708 | 746 | sonic hedgehog protein precursor [Griffithsia japonica] |
| comp6740_c0 | -2.4664 | 341 | Plant PEC family metallothionein |
| comp1647_c0 | -2.445 | 693 | deoxyribodipyrimidine photolyase family protein [Oceanibaculum indicum P24] |
| comp580_c0 | -2.4119 | 339 | hypothetical protein SELMODRAFT_165786 [Selaginella moellendorffii] |
| comp2692_c0 | -2.4 | 319 | 60s Acidic ribosomal protein |
| comp25297_c0 | -2.396 | 340 | WASP-interacting protein VRP1/WIP, contains WH2 domain |
| comp167449_c0 | -2.3877 | 204 | Trypsin |
| comp3821_c0 | -2.3669 | 328 | Sex peptide (SP) family//Protein of unknown function (DUF1687) |
| comp11576_c0 | -2.3665 | 625 | RRM domain-containing RNA-binding protein [Sphaerochaeta pleomorpha str. Grapes] |
| comp3933_c0 | -2.353 | 202 | Transcription factor TFIIB repeat |
| comp7894_c0 | -2.3522 | 743 | nucleolar RNA binding protein [Micromonas sp. RCC299] |
| comp1820_c0 | -2.3466 | 293 | hypothetical protein BN159_6424 [Streptomyces davawensis JCM 4913] |
| comp103228_c0 | -2.3314 | 363 | phosphoenolpyruvate/phosphate translocator [Galdieria sulphuraria] |
| comp8564_c0 | -2.33 | 429 | tRNA Pseudouridine synthase II, C terminal |
| comp2196_c0 | -2.3224 | 287 | unnamed protein product [Vitis vinifera] |
| comp8242_c0 | -2.3159 | 812 | hypothetical protein CAOG_00072 [Capsaspora owczarzaki ATCC 30864] |
| comp93170_c0 | -2.299 | 263 | DEAD box helicase [Ectocarpus siliculosus] |
| comp7082_c0 | -2.2955 | 1259 | hypothetical protein BN159_6424 [Streptomyces davawensis JCM 4913] |
| comp9163_c0 | -2.2892 | 519 | Tetraspanin family |
| comp4011_c0 | -2.2864 | 756 | Phycobilisome rod-core linker polypeptide [Pyropia yezoensis] |
| comp8876_c0 | -2.2809 | 751 | dhm exonuclease [Ectocarpus siliculosus] |
| comp11844_c0 | -2.2708 | 645 | hypothetical protein Echvi_3810 [Echinicola vietnamensis DSM 17526] |
| comp3634_c0 | -2.2612 | 748 | deoxyribodipyrimidine photolyase family protein [Oceanibaculum indicum P24] |
| comp168418_c0 | -2.2464 | 295 | PREDICTED: GTP-binding protein TypA/BipA homolog [Glycine max] |
| comp7535_c0 | -2.2419 | 566 | putative auxin-responsive protein [Arabidopsis thaliana] |
| comp65666_c0 | -2.232 | 229 | hypothetical protein GUITHDRAFT_117501 [Guillardia theta CCMP2712] |
| comp7018_c0 | -2.2124 | 292 | Serine/threonine protein kinase |
| comp2180_c0 | -2.2121 | 488 | Actin regulatory protein (Wiskott-Aldrich syndrome protein) |
| comp102276_c0 | -2.2105 | 472 | GUCT (NUC152) domain |
| comp42540_c0 | -2.2055 | 363 | Type III secretion needle MxiH like |
| comp10840_c0 | -2.2021 | 965 | predicted protein [Micromonas sp. RCC299] |
| comp12080_c0 | -2.2004 | 1127 | hypothetical protein Oter_3897 [Opitutus terrae PB90-1] |
| comp12580_c0 | -2.1854 | 1214 | Receptor of activated protein kinase C 1A, component of 40S small ribosomal subunit [Ostreococcus lucimarinus CCE9901] |
| comp9041_c0 | -2.181 | 721 | SAR DNA-binding protein-1-garden pea (ISS) [Ostreococcus tauri] |
| comp12853_c0 | -2.1793 | 2016 | ribose-phosphate pyrophosphokinase [Thermodesulfovibrio yellowstonii DSM 11347] |
