# Supplementary material for: Comparative transcriptome profiling of Pyropia yezoensis (Ueda) M.S. Hwang & H.G. Choi in response to temperature stresses
Source: BMC Genomics. 2015 Jun 17;16(1):463. doi: 10.1186/s12864-015-1586-1 (PMC4470342; doi:10.1186/s12864-015-1586-1)
Supplement: Additional file 10: Table S10. — GO enrichment analysis of (down and up)-regulated genes in HT compared with NT. [file 12864_2015_1586_MOESM10_ESM.docx]

Table S10 GO enrichment analysis of (down/up)-regulated genes in HT compared with NT

GO enrichment analysis of the down-regulated genes in HT compared with NT

| GO_accession | Description | Corrected_pValue | DEG_item | Bg_item |
| --- | --- | --- | --- | --- |
| **Biological process** |  | | | |
| GO:0022613 | ribonucleoprotein complex biogenesis | 1.52E-07 | 89 | 545 |
| GO:0042254 | ribosome biogenesis | 1.52E-07 | 89 | 545 |
| GO:0071843 | cellular component biogenesis at cellular level | 2.36E-07 | 94 | 591 |
| GO:0009066 | aspartate family amino acid metabolic process | 6.36E-07 | 36 | 114 |
| GO:0044085 | cellular component biogenesis | 3.89E-06 | 104 | 709 |
| GO:0071841 | cellular component organization or biogenesis at cellular level | 1.16E-05 | 119 | 870 |
| GO:0071840 | cellular component organization or biogenesis | 7.32E-05 | 124 | 946 |
| GO:0006555 | methionine metabolic process | 0.000103 | 12 | 22 |
| GO:0006412 | translation | 0.000109 | 103 | 749 |
| GO:0009058 | biosynthetic process | 0.000115 | 270 | 2342 |
| GO:0019752 | carboxylic acid metabolic process | 0.000148 | 93 | 607 |
| GO:0006520 | cellular amino acid metabolic process | 0.000201 | 77 | 466 |
| GO:0046459 | short-chain fatty acid metabolic process | 0.000268 | 12 | 25 |
| GO:0043436 | oxoacid metabolic process | 0.000318 | 93 | 614 |
| GO:0006082 | organic acid metabolic process | 0.000318 | 93 | 616 |
| GO:0006541 | glutamine metabolic process | 0.000513 | 9 | 18 |
| GO:0009987 | cellular process | 0.000745 | 476 | 4729 |
| GO:0044249 | cellular biosynthetic process | 0.001181 | 251 | 2215 |
| GO:0009308 | amine metabolic process | 0.001246 | 30 | 127 |
| GO:0042180 | cellular ketone metabolic process | 0.001609 | 94 | 652 |
| GO:0006006 | glucose metabolic process | 0.001609 | 23 | 79 |
| GO:0000096 | sulfur amino acid metabolic process | 0.002881 | 12 | 30 |
| GO:0009086 | methionine biosynthetic process | 0.002902 | 6 | 8 |
| GO:0009064 | glutamine family amino acid metabolic process | 0.003232 | 17 | 60 |
| GO:0006790 | sulfur compound metabolic process | 0.00381 | 23 | 92 |
| GO:0043648 | dicarboxylic acid metabolic process | 0.004274 | 17 | 64 |
| GO:0051790 | short-chain fatty acid biosynthetic process | 0.004609 | 6 | 9 |
| GO:0044237 | cellular metabolic process | 0.005281 | 385 | 3743 |
| GO:0044267 | cellular protein metabolic process | 0.007709 | 162 | 1374 |
| GO:0006094 | gluconeogenesis | 0.01283 | 15 | 47 |
| GO:0006531 | aspartate metabolic process | 0.014845 | 8 | 16 |
| GO:0019319 | hexose biosynthetic process | 0.016837 | 15 | 48 |
| GO:0046364 | monosaccharide biosynthetic process | 0.016837 | 15 | 48 |
| GO:0019318 | hexose metabolic process | 0.017628 | 24 | 102 |
| GO:0006007 | glucose catabolic process | 0.018521 | 19 | 71 |
| GO:0019320 | hexose catabolic process | 0.018521 | 19 | 71 |
| GO:0046365 | monosaccharide catabolic process | 0.019597 | 19 | 72 |
| GO:0009165 | nucleotide biosynthetic process | 0.019597 | 23 | 115 |
| GO:0009067 | aspartate family amino acid biosynthetic process | 0.019675 | 12 | 35 |
| GO:0000097 | sulfur amino acid biosynthetic process | 0.020122 | 6 | 11 |
| GO:0005996 | monosaccharide metabolic process | 0.020635 | 24 | 104 |
| GO:1901293 | nucleoside phosphate biosynthetic process | 0.027684 | 23 | 117 |
| GO:0016052 | carbohydrate catabolic process | 0.03046 | 19 | 78 |
| GO:0044724 | single-organism carbohydrate catabolic process | 0.03046 | 19 | 78 |
| GO:0072522 | purine-containing compound biosynthetic process | 0.03493 | 20 | 102 |
| GO:0009156 | ribonucleoside monophosphate biosynthetic process | 0.03493 | 9 | 26 |
| GO:0009161 | ribonucleoside monophosphate metabolic process | 0.03493 | 9 | 26 |
| GO:0009126 | purine nucleoside monophosphate metabolic process | 0.038072 | 7 | 19 |
| GO:0009127 | purine nucleoside monophosphate biosynthetic process | 0.038072 | 7 | 19 |
| GO:0009167 | purine ribonucleoside monophosphate metabolic process | 0.038072 | 7 | 19 |
| GO:0009168 | purine ribonucleoside monophosphate biosynthetic process | 0.038072 | 7 | 19 |
| GO:0044281 | small molecule metabolic process | 0.038511 | 136 | 1119 |
| GO:0008152 | metabolic process | 0.038836 | 461 | 4722 |
| GO:0008652 | cellular amino acid biosynthetic process | 0.041295 | 31 | 184 |
| GO:0006164 | purine nucleotide biosynthetic process | 0.041295 | 19 | 96 |
| **Cellular component** | | | | |
| GO:0044444 | cytoplasmic part | 1.52E-07 | 169 | 1215 |
| GO:0005840 | ribosome | 1.88E-07 | 92 | 571 |
| GO:0005737 | cytoplasm | 3.93E-07 | 205 | 1573 |
| GO:0030529 | ribonucleoprotein complex | 2.21E-06 | 97 | 638 |
| GO:0009507 | chloroplast | 6.07E-06 | 42 | 197 |
| GO:0009536 | plastid | 1.62E-05 | 44 | 218 |
| GO:0044435 | plastid part | 0.000304 | 28 | 112 |
| GO:0043228 | non-membrane-bounded organelle | 0.000318 | 120 | 937 |
| GO:0043232 | intracellular non-membrane-bounded organelle | 0.000318 | 120 | 937 |
| GO:0044434 | chloroplast part | 0.000429 | 26 | 105 |
| GO:0009535 | chloroplast thylakoid membrane | 0.000523 | 12 | 34 |
| GO:0055035 | plastid thylakoid membrane | 0.000523 | 12 | 34 |
| GO:0009534 | chloroplast thylakoid | 0.000937 | 12 | 35 |
| GO:0031976 | plastid thylakoid | 0.000937 | 12 | 35 |
| GO:0005622 | intracellular | 0.001463 | 309 | 2905 |
| GO:0043226 | organelle | 0.002428 | 235 | 2162 |
| GO:0043229 | intracellular organelle | 0.002428 | 235 | 2162 |
| GO:0031984 | organelle subcompartment | 0.002871 | 14 | 49 |
| GO:0044424 | intracellular part | 0.003074 | 289 | 2717 |
| GO:0042720 | mitochondrial inner membrane peptidase complex | 0.007654 | 4 | 5 |
| GO:0005623 | cell | 0.019452 | 319 | 3136 |
| GO:0044464 | cell part | 0.019452 | 319 | 3136 |
| GO:0042651 | thylakoid membrane | 0.025089 | 14 | 58 |
| **Molecular function** | | | | |
| GO:0003735 | structural constituent of ribosome | 1.02E-05 | 76 | 500 |
| GO:0036094 | small molecule binding | 1.62E-05 | 188 | 1414 |
| GO:0000166 | nucleotide binding | 4.38E-05 | 177 | 1330 |
| GO:1901265 | nucleoside phosphate binding | 4.38E-05 | 177 | 1330 |
| GO:0035639 | purine ribonucleoside triphosphate binding | 0.000113 | 149 | 1093 |
| GO:0001883 | purine nucleoside binding | 0.000256 | 149 | 1103 |
| GO:0032550 | purine ribonucleoside binding | 0.000256 | 149 | 1103 |
| GO:0032549 | ribonucleoside binding | 0.000271 | 149 | 1108 |
| GO:0032555 | purine ribonucleotide binding | 0.000271 | 149 | 1108 |
| GO:0032553 | ribonucleotide binding | 0.000318 | 150 | 1122 |
| GO:0017076 | purine nucleotide binding | 0.00033 | 149 | 1112 |
| GO:0001882 | nucleoside binding | 0.000346 | 149 | 1116 |
| GO:0043168 | anion binding | 0.000346 | 166 | 1267 |
| GO:0097159 | organic cyclic compound binding | 0.003063 | 286 | 2618 |
| GO:1901363 | heterocyclic compound binding | 0.003063 | 286 | 2618 |
| GO:0000287 | magnesium ion binding | 0.003782 | 14 | 45 |
| GO:0005525 | GTP binding | 0.004274 | 39 | 214 |
| GO:0019001 | guanyl nucleotide binding | 0.006679 | 39 | 220 |
| GO:0032561 | guanyl ribonucleotide binding | 0.006679 | 39 | 220 |
| GO:0008964 | phosphoenolpyruvate carboxylase activity | 0.007654 | 4 | 4 |
| GO:0005198 | structural molecule activity | 0.00937 | 88 | 732 |
| GO:0008172 | S-methyltransferase activity | 0.013628 | 6 | 12 |
| GO:0016830 | carbon-carbon lyase activity | 0.014033 | 17 | 65 |
| GO:0016741 | transferase activity, transferring one-carbon groups | 0.016935 | 27 | 135 |
| GO:0005524 | ATP binding | 0.019437 | 123 | 962 |
| GO:0004648 | O-phospho-L-serine:2-oxoglutarate aminotransferase activity | 0.023238 | 3 | 3 |
| GO:0004345 | glucose-6-phosphate dehydrogenase activity | 0.023553 | 4 | 5 |
| GO:0032559 | adenyl ribonucleotide binding | 0.028252 | 123 | 973 |
| GO:0016462 | pyrophosphatase activity | 0.028252 | 73 | 525 |
| GO:0019238 | cyclohydrolase activity | 0.028986 | 5 | 9 |
| GO:0030554 | adenyl nucleotide binding | 0.031789 | 123 | 977 |
| GO:0016818 | hydrolase activity, acting on acid anhydrides, in phosphorus-containing anhydrides | 0.032981 | 74 | 534 |
| GO:0004611 | phosphoenolpyruvate carboxykinase activity | 0.034865 | 4 | 5 |
| GO:0016829 | lyase activity | 0.034915 | 30 | 172 |
| GO:0017111 | nucleoside-triphosphatase activity | 0.038072 | 70 | 507 |
| GO:0016887 | ATPase activity | 0.041194 | 44 | 277 |

DEG_item means the number of DEGs in related to this GO function.

Bg_item means the number of all genes in this GO function.
