# Supplementary material for: Comparative transcriptome profiling of Pyropia yezoensis (Ueda) M.S. Hwang & H.G. Choi in response to temperature stresses
Source: BMC Genomics. 2015 Jun 17;16(1):463. doi: 10.1186/s12864-015-1586-1 (PMC4470342; doi:10.1186/s12864-015-1586-1)
Supplement: Additional file 11: Table S11. — Expression abundance of HSP genes in the eight samples. [file 12864_2015_1586_MOESM11_ESM.docx]

Table S11 Expression abundance of HSP genes in the eight samples

|  | RPKM | | | | | | | |  |
| --- | --- | --- | --- | --- | --- | --- | --- | --- | --- |
| Gene_Id | NT_1 | NT_2 | HT_1 | HT_2 | CS_1 | CS_2 | FS_1 | FS_2 | NR Description |
| comp9816_c0 | 3247.345551 | 2578.2458 | 4911.873 | 3146.651 | 3559.996 | 3575.496 | 1776.23784 | 1917.50212 | putative heat shock protein 70 |
| comp11203_c0 | 1690.353598 | 1285.994 | 1461.946 | 858.5145 | 2069.229 | 2126.202 | 666.210244 | 907.332366 | putative heat shock protein 90 |
| comp2735_c0 | 978.5030969 | 644.33715 | 4959.063 | 3482.607 | 569.0478 | 386.2106 | 427.645884 | 342.207049 | heat shock protein 70 |
| comp6578_c0 | 888.9401194 | 674.76797 | 1411.68 | 828.4093 | 1254.005 | 1348.833 | 423.254951 | 610.276574 | putative heat shock protein 70-2 |
| comp13157_c0 | 811.6916061 | 719.72604 | 912.712 | 757.2013 | 624.1142 | 479.1044 | 576.832903 | 346.814203 | heat shock cognate 70, partial |
| comp2728_c0 | 801.9710009 | 534.87217 | 4074.906 | 2655.227 | 450.6783 | 312.4979 | 363.460433 | 267.723413 | heat shock protein 70 |
| comp2752_c0 | 615.9597194 | 559.86604 | 672.2741 | 550.1542 | 403.8151 | 313.6805 | 404.396114 | 251.298788 | Protein HSP-4 |
| comp6347_c0 | 502.6006928 | 417.03778 | 459.9209 | 285.038 | 507.6601 | 673.2686 | 197.464207 | 141.16949 | chaperonin 60, mitochondrial |
| comp13107_c0 | 483.0748794 | 384.57182 | 433.1078 | 389.2238 | 326.1714 | 243.0461 | 312.113027 | 195.284816 | heat shock protein 70 |
| comp3278_c0 | 394.8655027 | 278.66716 | 1661.425 | 1362.901 | 185.4902 | 116.6771 | 151.752064 | 89.892549 | Heat Shock Protein 90, endoplasmic reticulum |
| comp5512_c0 | 383.8689108 | 422.3814 | 256.5513 | 206.7052 | 263.3414 | 317.6433 | 377.384607 | 256.78548 | HSP90 co-chaperone p23 |
| comp12358_c0 | 260.6194889 | 183.56931 | 104.5789 | 108.0366 | 129.5204 | 170.9956 | 146.077948 | 111.408642 | Hsc70/Hsp90-organizing protein HOP |
| comp9949_c0 | 254.566059 | 206.68879 | 178.9983 | 114.9916 | 249.3618 | 328.0756 | 99.0676782 | 101.015697 | Heat shock protein 90 |
| comp13402_c0 | 244.4939272 | 200.75925 | 242.4397 | 162.648 | 379.6252 | 411.7431 | 157.506275 | 199.191238 | heat shock protein 70 |
| comp10260_c0 | 242.1366623 | 205.68008 | 211.2981 | 174.9899 | 376.7021 | 385.2027 | 148.318559 | 196.109064 | heat shock protein 70 |
| comp12212_c0 | 225.6485458 | 168.10246 | 103.708 | 97.15045 | 98.43161 | 144.1288 | 116.765731 | 77.4213219 | 60 kDa heat shock protein, CPN60 protein |
| comp13049_c0 | 213.6313492 | 172.51524 | 209.5002 | 154.1994 | 221.1988 | 261.051 | 126.552259 | 149.019601 | HSP91 (Heat shock protein 91), putative |
| comp30324_c0 | 204.2531688 | 160.46489 | 79.59768 | 57.11143 | 111.9325 | 159.8633 | 82.3966211 | 27.4671572 | chaperonin Cpn60 |
| comp7933_c0 | 157.5953413 | 123.39144 | 65.75464 | 42.99273 | 80.53331 | 114.6836 | 63.7991881 | 19.8605963 | chaperonin GroEL |
| comp12891_c0 | 134.7230406 | 108.97315 | 418.6648 | 290.4044 | 80.79041 | 53.14943 | 75.181713 | 45.3271638 | Molecular chaperones GRP170/SIL1, HSP70 superfamily |
| comp38018_c0 | 96.58796581 | 83.845135 | 52.69089 | 51.69835 | 45.2738 | 62.96039 | 51.4183425 | 19.2830135 | groES, HSPE1 |
| comp11939_c0 | 92.80527114 | 82.273127 | 68.14466 | 63.24648 | 99.23806 | 98.07332 | 112.384426 | 130.067379 | Heat shock protein STI |
| comp9713_c0 | 83.87932448 | 46.847975 | 80.68089 | 74.76471 | 133.3947 | 136.6088 | 29.3960403 | 67.1878105 | Chaperone HSP104 and related ATP-dependent Clp proteases |
| comp10631_c0 | 69.32530807 | 44.233723 | 1407.235 | 671.3587 | 200.7628 | 249.3011 | 23.0185986 | 19.2297059 | heat shock protein Hsp20 |
| comp10288_c0 | 68.46570929 | 54.038226 | 41.40424 | 81.94726 | 27.04891 | 19.56188 | 10.1092016 | 18.5346274 | Heat shock protein 9/12 |
| comp40570_c0 | 37.56170553 | 31.643948 | 37.91528 | 30.46879 | 24.47414 | 25.57212 | 22.4190876 | 13.187559 | groES, HSPE1 |
| comp11438_c0 | 36.5518321 | 42.376839 | 54.24354 | 61.04099 | 47.21043 | 37.4885 | 39.4923007 | 33.9310103 | heat shock protein 70c |
| comp34823_c0 | 31.88534225 | 24.071037 | 29.87501 | 19.81377 | 25.86495 | 22.51977 | 20.3935454 | 16.6432733 | Chaperone HSP104 and related ATP-dependent Clp proteases |
| comp7379_c0 | 23.05092845 | 24.725142 | 23.37145 | 22.44784 | 33.79853 | 26.15959 | 22.159328 | 21.3398683 | Molecular chaperones GRP78/BiP/KAR2, HSP70 superfamily |
| comp165532_c0 | 17.46270407 | 15.928237 | 8.311083 | 5.76504 | 11.35535 | 20.42491 | 10.882219 | 7.80726888 | mitochondrial grpE-type co-chaperone of the HSP70 system |
| comp11281_c0 | 12.50479521 | 15.593338 | 7.441489 | 6.063625 | 22.54771 | 28.49969 | 13.8729229 | 10.4541183 | heat shock protein 70 |
| comp101925_c0 | 12.35991812 | 16.326443 | 15.20041 | 13.6729 | 9.311383 | 7.516366 | 6.20759625 | 6.82875785 | molecular chaperone Hsp33 |
| comp163272_c0 | 9.151507322 | 3.4985234 | 6.145717 | 7.272819 | 11.08498 | 8.753532 | 7.20524565 | 8.12947363 | Hsp33 protein |
| comp43947_c0 | 5.461257566 | 4.7323022 | 3.117393 | 5.797175 | 7.309661 | 4.144184 | 6.18510496 | 6.70092482 | putative heat shock protein 70-2 |
| comp7983_c0 | 3.892119876 | 20.219671 | 20.20387 | 23.69769 | 17.67047 | 19.63933 | 12.230701 | 6.04132168 | HSP70 |
| comp2560_c0 | 1.849876881 | 9.4219558 | 3.279148 | 13.38351 | 7.208725 | 10.48669 | 5.96612944 | 2.82790562 | heat shock protein 70 B2, partial |

RPKM: 0.1~3.75 genes with low expression abundance

RPKM: 3.75~15 genes with medium expression abundance

RPKM: >15 genes with high expression abundance
