# Supplementary material for: Comparative transcriptome profiling of Pyropia yezoensis (Ueda) M.S. Hwang & H.G. Choi in response to temperature stresses
Source: BMC Genomics. 2015 Jun 17;16(1):463. doi: 10.1186/s12864-015-1586-1 (PMC4470342; doi:10.1186/s12864-015-1586-1)
Supplement: Additional file 12: Table S12. — Primers used in qRT-PCR for validating differentially expressed genes. [file 12864_2015_1586_MOESM12_ESM.docx]

Table S12 Primers used in qRT-PCR for validating differentially expressed genes

| Gene ID | Description | Sense primers (5'-3') | Antisense primers (5'-3') |
| --- | --- | --- | --- |
| comp3278_c0 | Heat Shock Protein 90 | GGCGACTCCTCCAACCTTATC | GCACCGCTCTCCCACACAT |
| comp11158_c0 | putative delta-5 fatty acid desaturase | TCCACGGCAACGTCTATGAC | CTTGCTCGTGAACGGATGGTA |
| comp11861_c0 | delta-6 fatty acid desaturase | TTAAACAAGCTCGGCGTCAC | GCGTGGTCTTTACCGTAGGC |
| comp12448_c0 | predicted protein | GACCGAGCGCCTTCTTGAG | GGACCGGGACTACGGCATCT |
| comp12584_c0 | fatty acid desaturase | AGAAGATTTCGTCCCGCTCAT | CACGCCCTCAACTCCTTTATT |
| comp10631_c0 | heat shock protein Hsp20 | AGTCACCTGCGGGTCCTTGT | GACGCGGATGTCGAGTCCA |
| comp8549_c0 | Ycf46 | ACGTAGCAGAGGCAGCATCCA | CGGTATTCCTTCGCCCAAA |
|  | *ACT3* | CAAGCAGAAGGGCATCAT | CCGAGTAGAAAGCGTGGT |
|  | *eIF4a* | GCTTTCTGTCTGGACGAGG | TCTTCACAAGGATGCGGAT |
